# Supplementary material for: Ion mobility mass spectrometry and molecular dynamics simulations unravel the conformational stability of zinc metallothionein-2 species
Source: Chem Commun (Camb). 2023 Mar 24;59(30):4471–4. doi: 10.1039/d2cc06559b (PMC10089061; doi:10.1039/d2cc06559b)
Supplement: CC-059-D2CC06559B-s001 [file CC-059-D2CC06559B-s001.pdf]

Electronic Supplementary Material (ESI) for ChemComm.
This journal is © The Royal Society of Chemistry 2023

Electronic Supporting information

Ion mobility mass spectrometry and molecular dynamics simulations unravel the conformational stability of human metallothionein-2 species

Manuel David Peris-Díaz[ab*], Alexey Barkhanskiy[b], Ellen Liggett[b], Perdita Barran[b*] Artur Krężel[a*]

[a] *Department of Chemical Biology, Faculty of Biotechnology, University of Wrocław, F. Joliot-Curie 14a, 50-383 Wrocław, Poland*
[b] *Michael Barber Centre for Collaborative Mass Spectrometry, Manchester Institute of Biotechnology, 131 Princess Street, Manchester, M1 7DN, United Kingdom*

TABLE OF CONTENTS

Materials S2
Expression and purification of metallothioneins..... S2
Mass spectrometry. S3
Computational studies..... S6
Figure S1..... S11
Figure S2..... S12
Figure S3..... S12
Figure S4..... S13
Figure S5..... S14
Figure S6..... S16
Figure S7..... S17
Figure S8..... S18
Figure S9..... S19
Figure S10..... S20
Figure S11..... S21
Figure S12..... S22
Figure S13..... S23
Figure S14..... S24
Figure S15..... S20
Figure S16..... S21
Figure S17..... S22
Figure S18..... S23
Figure S19..... S24
Figure S20..... S30
Figure S21..... S31
Figure S22..... S32
Figure S23..... S33

Figure S24 .... S34
Figure S25 .... S35
Table S1 .... S36
Table S2 .... S37
REFERENCES S38

EXPERIMENTAL SECTION

Materials. The following reagents: $ZnSO_4 \cdot 7H_2O$, 4-(2-pyridylazo)resorcinol (PAR), $(NH_4)_2CO_3$, tris(hydroxymethyl)aminomethane (Tris base) and 4-(2-hydroxyethyl)-1 piperazineethanesulfonic acid (HEPES), mass spectrometry grade methanol, tris(2carboxyethyl)phosphine hydrochloride (TCEP), ammonium acetate (AmAc) ethylenediamine-tetraacetic acid (EDTA), and mass spectrometry grade acetonitrile (ACN) were purchased from Sigma-Aldrich. Resin Chelex 100 was acquired from Bio-Rad and 98% hydrochloric acid (HCl) was purchased from VWR Chemicals. Dithiothreitol (DTT) was purchased from Iris Biotech GmbH. Tryptone, LB broth, yeast extract, isopropyl-β-D-1-thiogalactopyranoside (IPTG), and SDS were from Lab Empire, NaCl, NaOH, glycerol, $KH_2PO_4 \cdot H_2O$, K_2HPO_4 from POCH (Gliwice Poland), pTYB21 vector and chitin resin were from New England BioLabs, and 5,5′-dithiobis-(2-nitrobenzoic acid) (DTNB) from TCI Europe N.V. was purchased from Sigma-Aldrich.

Expression and purification of metallothioneins. Expression vector (Addgene plasmid ID 105693) were transformed into BL21(DE3) *E. coli* cells and growth in culture medium (1.1% tryptone, 2.2% yeast extract, 0.45% glycerol, 1.3% K_2HPO_4, 0.38% KH_2PO_4) at 37°C until ~0.5 OD_{600}. Protein was induced by adding 0.1 mM IPTG to cells and overnight incubation at 20°C with vigorous shaking. The next steps were conducted at 4°C. Cells were centrifuged (4,000 × *g* for 10 min) and resuspended in 50 mL of cold buffer A (20 mM HEPES, pH 8.0, 500 mM NaCl, 1 mM EDTA, 1 mM TCEP). This was followed by sonicated for 30 min (1 min cycles) and centrifugation (20,000 × *g* for 15 min). The expressed protein was purified by a chitin resin. Briefly, after centrifugation, the supernatant was incubated overnight with 20 mL of chitin resin in buffer A, then washed with 50 ml of buffer A and cleavage by the addition of 100 mM DTT. The resin was incubated for 48 h at room temperature on a rocking bed. The eluted solution from the chitin column was acidified to pH ~ 2.5 with 7% HCl and concentrated using Amicon Ultra-4 Centrifugal Filter Units with a membrane cut-off of 3 kDa (Merck Millipore,

USA). The protein was then purified on a size exclusion chromatography SEC-70 gel filtration column (Bio-Rad) equilibrated with 10 mM HCl, then obtaining metal-free protein (apoMT2).[1] The identity of the eluted protein from SEC was confirmed by ESI-MS using a Bruker Maxis Impact (Bruker Daltonik GmbH, Bremen, Germany) calibrated with a commercial ESI-TOF Tuning mix (Sigma-Aldrich). Thiol concentration was determined spectrophotometrically using a DTNB assay[2], and the Zn^{2+} binding capacity was confirmed spectrophotometrically by Zn^{2+} and Cd^{2+} titrations.[3] To the collected fraction of purified thionein-2, 10 molar excess of $ZnSO_4$ was added under a nitrogen blanket, and the pH was adjusted to 8.6 with a 1 M Tris base. Samples were concentrated with Amicon Ultra-4 Centrifugal Filter Units with a membrane cut-off of *3* kDa (Merck Millipore, USA) and subsequently purified on an SEC-70 gel filtration column (Bio-Rad) equilibrated with 20 mM Tris-HCl buffer at pH 8.6. Concentrations of thiols and Zn^{2+} were determined spectrophotometrically using DTNB and PAR assays, respectively.[4]

Mass spectrometry

Nanoelectrospray ionization. All samples were prepared at 10-20 µM in 200 or 50 mM ammonium acetate (AmAc), pH 6.8 supplemented with 1 mM TCEP, and desalted using micro Bio-Spin 6 columns (Bio-Rad) prior to any experiment. The samples were then ionized from a borosilicate glass capillary (O.D. 1.2 mm, I.D. 0.9 mm, World Precision Instruments, Stevenage, UK) produced in-house using a Flaming/Brown P-1000 micropipette puller (Sutter Instrument Co., Novato, CA, USA). Ions were produced by applying a positive potential of 0.9-1.4 kV via a platinum wire (Goodfellow).

Linear travelling wave ion mobility mass spectrometry. Linear TW IM-MS experiments were carried out on a Synapt XS HDMS (Waters Corporation, Manchester, UK). Gentle source conditions were applied to prevent ion activation (source temperature 30ºC, cone voltage 20 V, source offset 1). All of the experiments were carried out in sensitivity mode to maximize ion transmission at the expense of peak resolution. Collision-induced unfolding (CIU) experiments were performed by mass-selection of ions of interest by the quadrupole mass analyzer and then by recording ion arrival time distributions under different trap collision energies (CE) in the 0-60 V range. Wave velocity and wave heights wave velocity and height were set up at 300 ms^{-1} and 20 V,

respectively. The helium cell and nitrogen traveling wave were operated at 200 and 75 mL·min^{-1}. To minimize ion activation, the trap DC bias was set up at 35 V. The mass spectra were calibrated using 2 µg·µL^{-1} NaI made up of 1:1 water:isopropanol. Ion activation energies were reported as laboratory frame energy (E_{lab}), accounting for the charge state of the mass-selected ion. Arrival time distributions were calibrated to $^{TW}CCS_{N2}$ using a TWIMS calibration procedure. Ubiquitin (bovine), cytochrome C (equine heart), and β-lactoglobulin (bovine milk) were purchased from Sigma-Aldrich and used as calibrants. The lyophilized powders were dissolved in either 200 or 50 mM AmAc and diluted to a 10 µM protein concentration. IM-MS data was recorded on three different days, and data were averaged. The literature CCS_{N2} values for the standards were obtained from A. P. France *et al.*[5]

Optimization of salt concentration for ESI-MS studies. Our calibration was based on protein calibrants dissolved in similar solutions conditions as for the subsequent analysis (50 mM AmAc, 1 mM TCEP). In contrast, Russell and coworkers reported a TW calibration based on denaturing calibrants.[6] Moreover, no differences in the CCSD were obtained when spraying the proteins at higher salt content, 200 mM AmAc (Figure S1). In addition, calibration of the TW cell with different wave heights and velocities yielded similar CCS values. To note, similar wave height and velocity as it was used in their studies were used here. Therefore, the CCS differences could be more likely attributed to these calibration differences.

Avoiding drastic pH drop is especially relevant for metallothioneins, where 20 thiolates coordinating Zn^{2+} ions can be easily protonated, resulting in Zn^{2+} dissociation. However, ammonium acetate solution does not constitute a buffer at neutral pH since the buffering properties of ammonium acetate are at pH 4.75 ± 1 and 9.25 ± 1, which corresponds to the acetic acid and ammonium pK_as.[7] During the desolvation process in the ESI plume, protonation of acetate generates acetic acid (in ESI positive mode) and favors the formation of a $[M + zH]^{z+}$ ions. While this process leads to inevitable acidification, likely close to the pK_a values of acetic acid, the higher the AmAc concentration, the lower the pH is shifted. For instance, a pH drop to 6.5 can be estimated when using 100 mM ammonium acetate as a solution.[7] Lowering the pH would, in turn, protonate Cys residues directly affecting the stability of the Zn^{2+}-binding sites.[8] Therefore, to mitigate the acidification process, the following experiments were mostly performed with 200 mM AmAc.

Cyclic travelling wave ion mobility mass spectrometry. Cyclic TW IMS experiments were performed on a Select Series Cyclic IMS instrument (Waters. Corporation, Manchester, UK). Samples were analyzed under similar source conditions as the Synapt XS. The TWIMS wave velocity and height were set up at 375 ms^{-1} and 20 V, respectively. The helium cell and IMS nitrogen gas flow rates were 150 and 45 $mL{\cdot}min^{-1}$.

Multipass cIM. Quadrupole-selected Zn_7MT2^{5+} ions (1298 *m/z*) were subjected to 1-3 passes around the cyclic device to obtain higher mobility resolving power. The trap, cyclic IM, and transfer cell were operated as gently as possible to minimize ion activation while obtaining a reasonable transmission. The ions were injected into the trap cell with an acceleration voltage of 5 V, and the post-trap bias was set up at 25 V since we could observe ion activation at standard values (45 V). The He cell bias was kept at 20 V. The ion packet from the trap cell is then injected into the cIM cell with a pre-array gradient and pre-array bias set up at 85 V, and 70 V, array offset of 25 V, and array entrance 10 V. The ions underwent 1 to 3 passes, with the wave velocity and height as abovementioned. Mobility separated ions are ejected from array to TOF analysis. Transfer collision energy was set to 10 V, to maximize the ion transmission without ion activation. Arrival time distributions (ATDs) were extracted in the [1295:1303] *m/z* range. The ATDs were deconvoluted by means CIUSuite 2 software.[9] The following parameters were used: maximum number of components = 10; peak amplitude = 0.05; peak overlap penalty mode = relaxed; expected fwhm were obtained similarly to Deslignière *et al.*[10] Briefly, a portion of ions from the convoluted ATD was selected and ejected to the pre-store while remaining ions ejected to the TOF. Then, those ions were reinjected from the pre-store to the array, separated, and the fwhm was calculated for the ATD recorded (1.50 ms). Slicing out the convoluted ATD to calculate fwhm provides accurate initial values for the peak modeling process. After one pass, a fwhm of 1.50 ms was calculated for the isolated slice. As the fwhm scales as the $\sqrt{n}$, where n is the number of passes for a peak with a single conformer, we could estimate the initial fwhm for 2 (2.12 ms) and 3 passes (2.59 ms). The resolving power was calculated as CCS/ΔCCS, where ΔCCS is the extracted fwhm of the mobility peak.

IMS-CA-IMS. To study the presence of different ion populations in the mass-selected Zn_7MT2^{5+}, we used a multistage IMS[2]. As above, we utilized the same parameters in the trap (5 V), post-trap bias (25 V) and helium cell (20 V) that minimize ion activation prior to the separation in the cIM device. A mobility-selected ion population or slice was

ejected and trapped in the pre-store array, while the remaining ions were ejected to the TOF. Then, the isolated ions were reinjected from the pre-store into the array. Unfolding of the mobility-selected ions was done by increasing both the pre-array gradient and the pre-array bias while keeping the voltage difference between these two parameters the same. Activated ions were subjected to one pass around the cyclic array and then ejected to the TOF. Worth comment is the need to perform two control experiments, named background ion signal and ion aging experiments.[11-12] The first one checks if the ions observed after reinjection from the pre-store are derived only from the isolated population. The second control experiment verifies the effect of time on the protein conformation. The ions are accumulated for a prolonged period of time in the array before separation.

CA-IMS-CA-IMS. This mode of operation is an extension to the IMS-CA-IMS with the difference that ions are also activated in the trap cell prior to the cIM.[11-12] The trap cell was used to activate all the ions before doing the IMS-CA-IMS.

MS data analysis. Data were analysed by means Masslynx v4.2 (Waters Corp., UK), ORIGAMI,[13] CIUSuite 2,[9] and Python 3.5 scripts.

Computational studies

Model building. The X-ray structure PDB ID 4MT2, which contained four Cd^{2+} and two Zn^{2+} ions, was selected as the initial structure. The initial Zn_7MT2 was obtained by replacing the Cd^{2+} with Zn^{2+} ions, and point mutations were done to match with the human MT2 sequence with the VMD mutator plugin.[14] All of the simulations were performed using the GROMACS 2018.4 software.[15] The AMBER FF19SB force field was used to model the protein, and derived parameters were used to describe cysteine-Zn^{2+} interactions.[16] Because of the lack of structural X-ray of NMR models for the partially Zn^{2+}-loaded MT2 species, $Zn_{4\text{-}6}$MT2 structures were obtained by well-tempered parallel-bias metadynamics (WT PB-MetaD).[3] Cluster analysis of the free energy minima obtained by WT PB-MetaD was used to obtain initial conformations for our studies. In particular, for Zn_6MT2, two configurations were included. In the first one, four Zn^{2+} are bound in the α-domain and two Zn^{2+} in the β-domain (αZn_4βZn_2MT2). In the second one, three Zn^{2+} are bound in each domain (αZn_3βZn_3MT2). For Zn_5MT2, one representative structure was extracted by cluster analysis from the basin obtained by WT PB-MetaD

($\alpha Zn_3\beta Zn_2MT2$). For Zn_4MT2, two configurations were used, $\alpha Zn_2\beta Zn_2MT2$ and $\alpha Zn_3\beta Zn_1MT2$.

Charge state distribution. To perform an MD simulation of $[M + zH]^{z+}$ that fully represents the experimental state(s) post nESI is non-trivial. The inclusion of water-water and water-protein proton transfer events cannot be captured by standard force fields[17] and would require the use of quantum mechanics (QM) treatments. Including ~ 2500 molecules in a QM part of a QM/MM scheme is computationally prohibitive nowadays. Here, we incorporated three alternative solutions to tackle this issue, namely: (i) a mobile Na^+ charge scheme in which all titratable residues were set up to their default pH 7.0 protonation state, except for the Cys residues, and Na^+ ions carried out the positive charges. Here, the Cys residues that had bound Zn^{2+} were modeled in the deprotonated form as this is how they are found experimentally in the X-ray structure and determined in this study by MS. In the partially Zn^{2+}-depleted and metal-free MT2 species, the free Cys residues were modeled as protonated in agreement with our MS data; (ii) a mobile Na^+/static H^+ charge scheme in which the negative charges (Asp and Glu residues) were neutralized while protonating the positive residues (Lys residues).[18] We should consider that Zn^{2+} carries a 2+ charge, each Cys-binding residue has a -1 charge, and a free Cys residue is neutral. In our case, metallothionein does not contain enough residues to distribute the *z* excess protons on the protein. In this approach, it is not fully correct to consider that the acidic sites are generally neutral since carboxylates $R\text{-}COO^-$ sites involved in salt bridges have been found.[18] Then, in all of the systems, several Na^+ were added to obtain a total system charge of 5+ and; (iii) a mobile H^+ approach that considers that the protons are highly mobile and the preferred proton-binding residues can change as the simulation, and therefore the protein structure evolves.[19-20] We used a recently released charge placement algorithm (ChargePlacer),[21] albeit modified to incorporate Zn^{2+}-binding residues in determining of the protonation pattern. The MD simulation was divided into multiple 50 ps NVT runs, and at the beginning of each simulation, the charge placement algorithm redistributed the protons along all of the titratable residues and all of the 20 Cys residues, independently if they had bound a Zn^{2+} ion. Briefly, the ChargePlacer algorithm finds the protonation pattern that minimizes the total energy of the system, which accounts for both Coulomb repulsion and proton affinity, resembling the approach described by Konermann.[19-20]

Gas-phase desolvation MD. Each protein system was solvated in a rhombic dodecahedron box with ~ 2500 TIP4P/2005 water molecules, since they provide more realistic results of the ESI droplet evaporation.[18] The three-site TIP3P water molecule is treated as a nonpolarizable molecule and exhibits a lower ~30% surface tension than the real water molecules. Here we employed the Na^+ mobile approach by which the aqueous droplet was charged by randomly replacing water molecules with excess Na^+ to obtain a system total charge of 16+. According to the charge residue mechanism (CRM) the maximum charge of positive charges that an ion can obtain is the Rayleigh limit (z_R).[22] For globular spherical protein, $z_R = 0.0778\ m^{1/2}$, where m is the molecular weight of the protein in Da units.[23] The value of z_R can deviate for proteins that are not structurally globular.[24] To account for any deviation, we employed a $2.5z_R$ excess of Na^+ ions in the initial droplets. MD runs with $z \sim z_R$ yielded similar final $[M+ zNa]^{z+}$ ions and z/z_R.

We carried out a pseudo-PBC approach in which the Coulomb and Lennard-Jones cutoffs were set to 300 nm, and the PBC box dimensions were set up to 900 nm^3. Although this approach uses periodic boundary conditions, the box size and cutoffs exclude interactions between PBC images. Each system was subjected to energy minimization by 10 000 steps of steepest descent minimization, followed by 10 ps NVT equilibration to 350 K by using the Nosé-Hoover thermostat with a coupling constant of 0.1 ps. The LINCS algorithm was used to constraint bonds involving hydrogen atoms to be able to use a 2 fs time step, and neighbor list was updated every 100 steps using the Verlet method. To simulate droplet desolvation, the MD simulations were split into multiple consecutive 250 ps length NVT runs. At the end of each window, water molecules and Na^+ and/or Zn^{2+} ions further than 30 Å from the center of mass of the protein were removed. The system was then recentered in the box, and the velocities reassigned from a Maxwell-Boltzmann distribution. This approach avoids evaporative cooling problems and speeds up the MD simulations as the number of particles is reduced. After 250 runs or 62.5 ns of NVT production at 350 K, the system was equilibrated to 500 K, and run for 5 ns to remove the last water molecules, also called "sticky" waters. Two independent runs were performed for each system, amounting to 0.9 µs of dynamics.

Collision cross section values were calculated every 250 ps using the trajectory method implemented in IMPACT software.[25] The simulations were analyzed using MDAnalysis 2.0[26-27], MDTraj 1.98[28], pytraj 2.0.5,[29] and in-house Python 3.5 scripts.

Gas-phase MD simulation. In contrast to the desolvation protocol, here, each protein system was first equilibrated in the presence of solvent molecules and then directly placed in pseudo-vacuum conditions. To compare it with our experimental data, the (i) mobile Na^+ charge scheme, (ii) mobile Na^+/static H^+ charge scheme, and a (iii) mobile H^+ approach were considered to capture the charge state distribution for the $[M + 5H]^{5+}$ ions. As above, we used a pseudo-PBC approach and ran each system for 100 ns at 298 K, and 798 K on three replicate runs for the (i) and (ii) approaches. While approaches (i) and (ii) were run for the seven protein systems, approach (iii) was run twice for 1500 ns at 298 K exclusively for the Zn_7MT2 structure. The cumulative simulation time approached 11.4 μs.

Simulated Annealing. To simulate protein unfolding as obtained by CIU experiments, structures obtained after gas-phase desolvation underwent a simulated annealing (SA) protocol with the pseudo-PBC approach. In the SA protocol the temperature was rise linearly from 298 to 798 K during 10 ns, we also compared results when applying a 100 ns run. Each system was run on three replicate runs. A total of 210 ns of SA were run. Data from three temperature ranges (300-350, 450-550, 750-850 K) was then extracted, and modeled using Gaussian kernel density estimation (KDE). Results are shown in Figure 4b.

Steered molecular dynamics (SMD) simulations. SMD was used to study the nonequilibrium unfolding dynamics of the protein systems obtained after gas-phase desolvation. Identical parameters as above were used for pseudo-PBC, temperature coupling, and bond parameters. The SMD computations were performed with GROMACS 2018.4 in combination with the PLUMED plugin.[30] Two different collective variables (CV) were considered: the end-to-end Met1(CA)-Ala61(CA) distance and the radius of gyration (R_g). The pulling force was optimized to be high enough to observe a linear dependence between the distance and the CV position while not producing high fluctuations in the force profile. In the first CV, three force constants (48, 95, and 190 $kcal \cdot mol^{-1} \cdot nm^{-2}$) with a pulling speed of 10 $Å \cdot ns^{-1}$ were used to produce extensions up to 50 Å so that Zn^{2+} dissociation does not occur, as observed during collision-induced unfolding experiments. The relationship between the pulling speed and rupture or unfolding force was also considered by using several pulling rates: 1, 10, and 100 $Å \cdot ns^{-1}$. The simulation time needed for each pulling speed was calculated as $(r_F - r_0)/k$, where r_F is the final distance, r_0 is the initial distance, and k is the pulling speed. Thus, 50, 5, or 0.5 ns were run for each pulling rate assayed, respectively. A pulling speed of 10 $Å \cdot ns^{-1}$

was chosen as it gave comparable unfolding forces as the lowest speed considered (1 Å·ns^{-1}) but saved computational time. In another set of SMD simulations, *R*g was employed as a CV. Initials trials established the maximum *R*g value needed to promote a conformational transition from a compact to an unfolded structure with intact Zn^{2+} binding sites. Thus, *R*g moved from an initial value of 11 Å to 18 Å. As above, three different force constants of 10, 25, and 50 kcal·mol^{-1}·nm^{-2} were used. Gaussian kernel density estimation (KDE) was used to model each force-CCS dataset and presented in Figure 4C. In total, 225 ns of SMD simulation time was considered for analysis. The RMSD, number of Zn─S bonds, number of hydrogen bonds (h-bonds), and number of salt bridges were computed by MDAnalysis 2.0[26-27] and Python 3.5 scripts. Statistical analysis of the forces and work were performed by using a two-tailed *t*-test with equal variances.

Well-Tempered Metadynamics (WT-MetaD) simulations. Metadynamics[31] simulations was used to estimate the free energy of unfolding dynamics of the protein systems obtained after gas-phase desolvation. To do so, a CV that described the distance between the center of mass between both protein domains was built. The temperature was set to a K_BT of 0.6 kcal/mol and a CV width of 0.5 Å was selected. Gaussian hills deposited every 500 time steps with an initial height of 0.6 kcal/mol and rescaled with a bias factor of 80. Such "aggressive" acceleration of the sampling ensures that all energy barriers are easily overcome at room temperature and provide a fast exploration of the conformational space, at expenses of convergence.[32] We used upper walls to avoid sampling unphysical states, that is when Zn^{2+} dissociation occurs. The free energy was reconstructed after the recrossing event, and three independent runs were considered for the analysis. The WT-MetaD were performed with GROMACS 2018.463 in combination with the PLUMED plugin.[30]

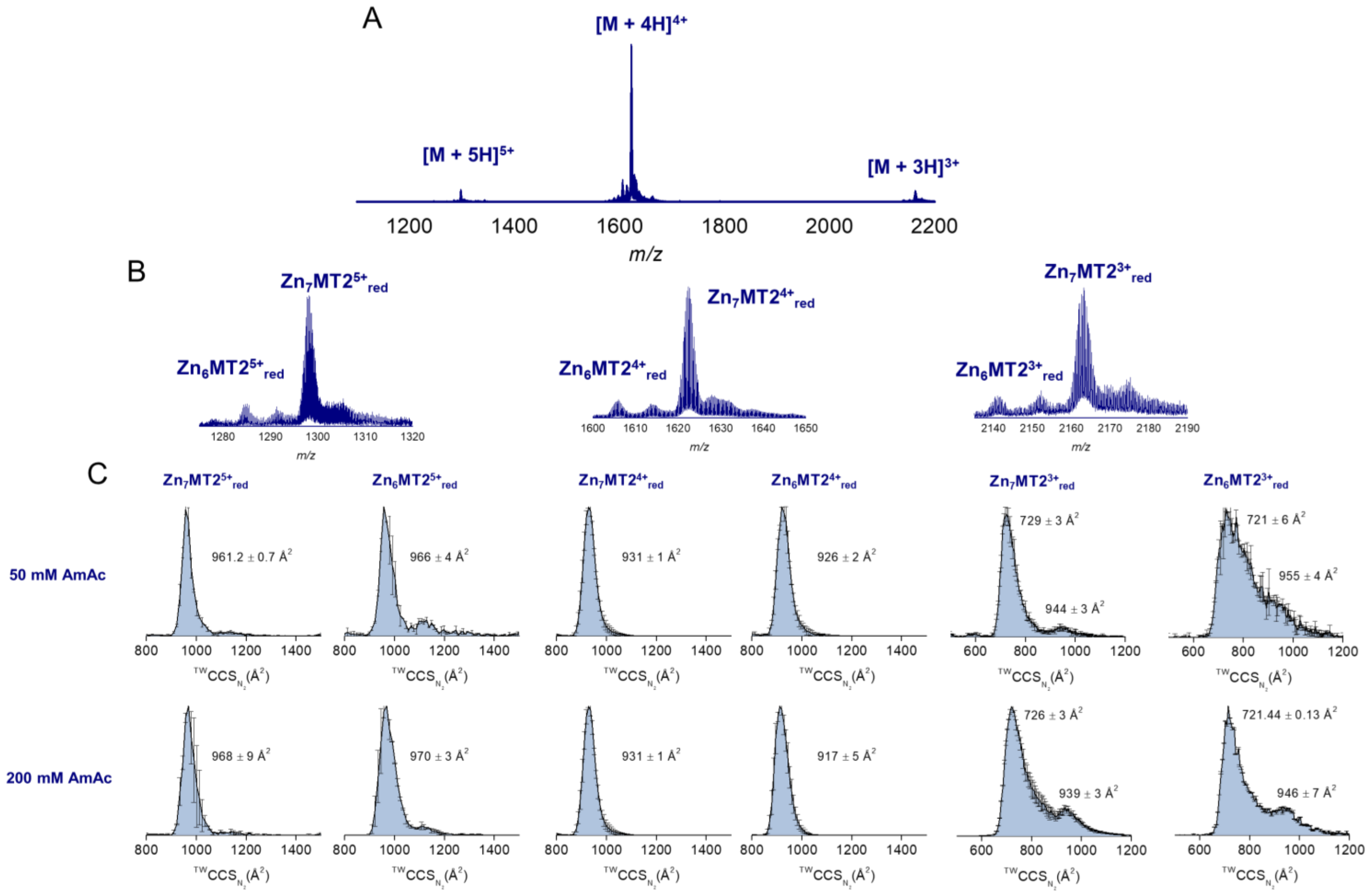

Figure S1. Native MS and CCS distributions for Zn_7MT2 (10 μM) sprayed under 50 and 200 mM AmAc supplemented with 1 mM TCEP. The CCS values were calculated from three replicates, and the error bars plot along the CCS axis.

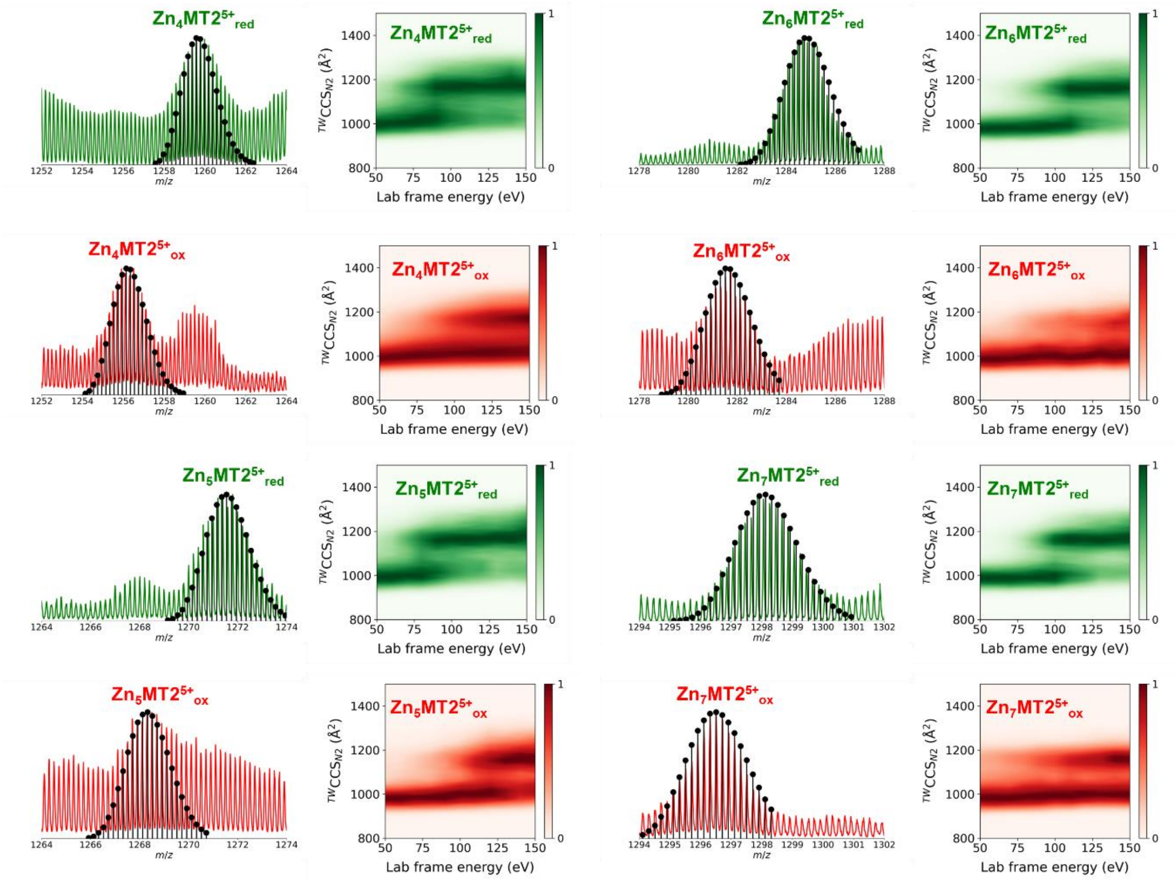

Figure S2. Native mass spectra and collision-induced unfolding (CIU) heat maps for the mass-selected 5+ ions of reduced and oxidized Zn_xMT2 (x = 4-7) sprayed from 50 mM ammonium acetate (pH 6.8) in the presence (reduced forms, “red”) and absence (oxidized, “ox”) of 1 mM pH neutralized TCEP (pH 7.4). Theoretical isotopic patterns are shown in black as stem plots, and the molecular formulas are shown in **Table S1**. Activation of the ions was performed in the trap cell prior to the IM cell by applying a linear collision energy ramp between 5-50 V with increments of 5 V.

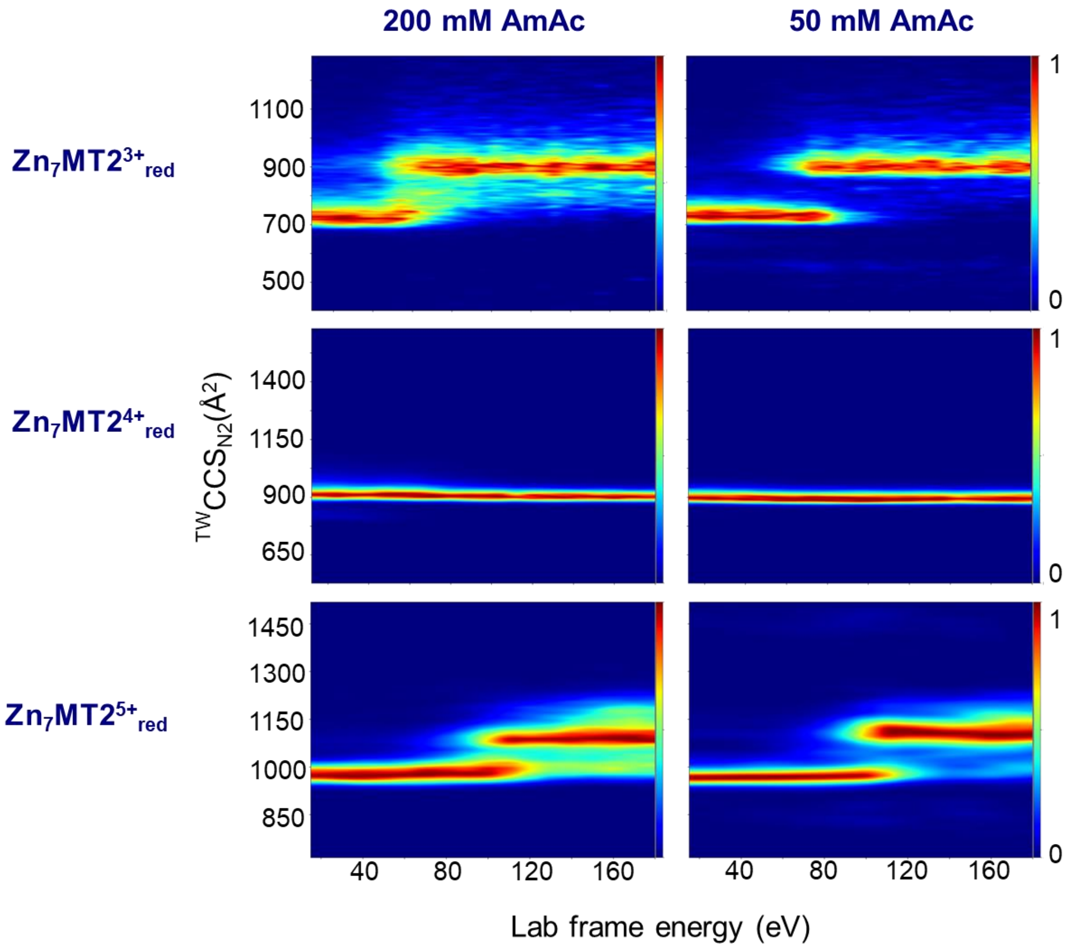

Figure S3. Collision induced unfolding (CIU) heat maps for $Zn_7MT2^{Z+}{}_{red}$ (z = 3-5) sprayed from 50 or 200 mM ammonium acetate (AmAc) supplemented with 1 mM TCEP.

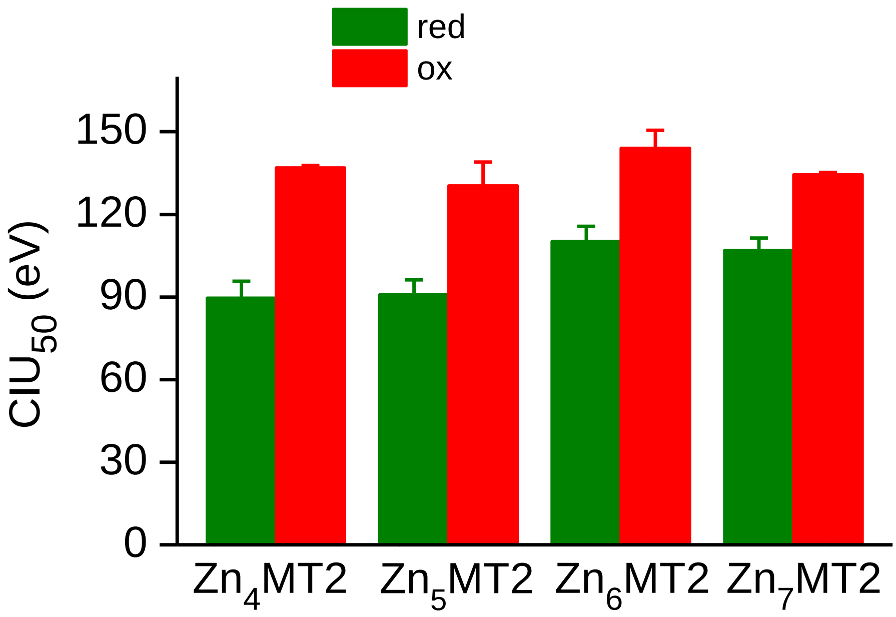

Figure S4. Gas-phase stabilities of $Zn_{4\text{-}7}MT2^{5+}$ ions measured as CIU_{50}, which in our case refers to the energy required to promote a conformational transition of 50 % of the ions from a compact to an extended conformation. CIU_{50} was calculated by fitting the collision-cross section distributions along the collision energies applied. Red and ox refer to reduced and oxidized ions, respectively.

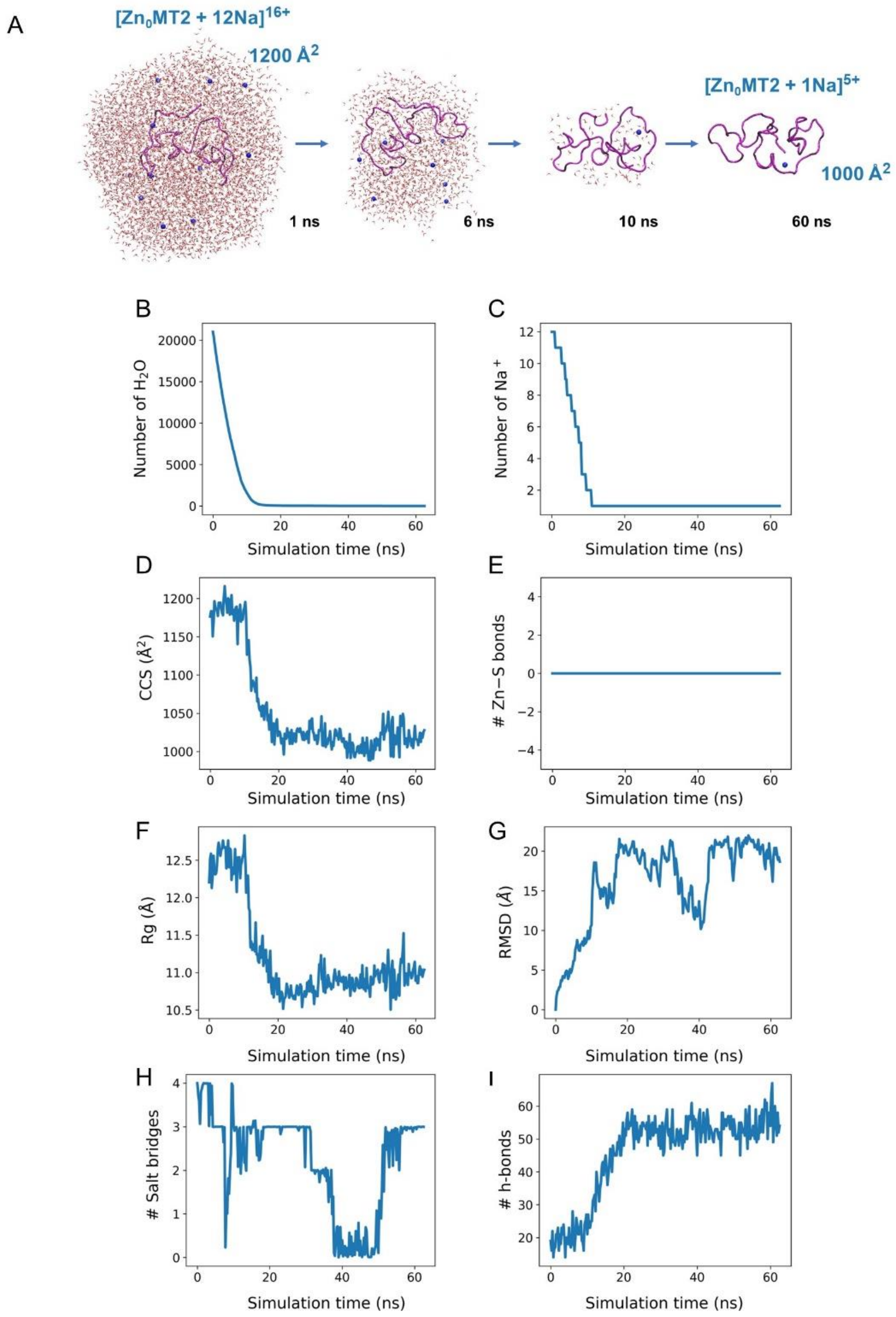

Figure S5. Gas-phase MD simulations of the electrospray ionization process of aqueous nanodroplet containing Zn_0MT2 and Na^+ to achieve a 16+ overall charge. (A) Snapshots of the desolvation process at different frames. Na^+ is represented by a blue sphere, the protein backbone is shown in magenta, and the oxygen atoms from solvent molecules are shown in red. The number of water molecules (B), Na+ ions (C), CCS values (D), number of Zn–S bonds (E), the radius of gyration (F), root-mean-square deviation (G), number of salt bridges (H) and hydrogen bonds (I) were monitored throughout the desolvation.

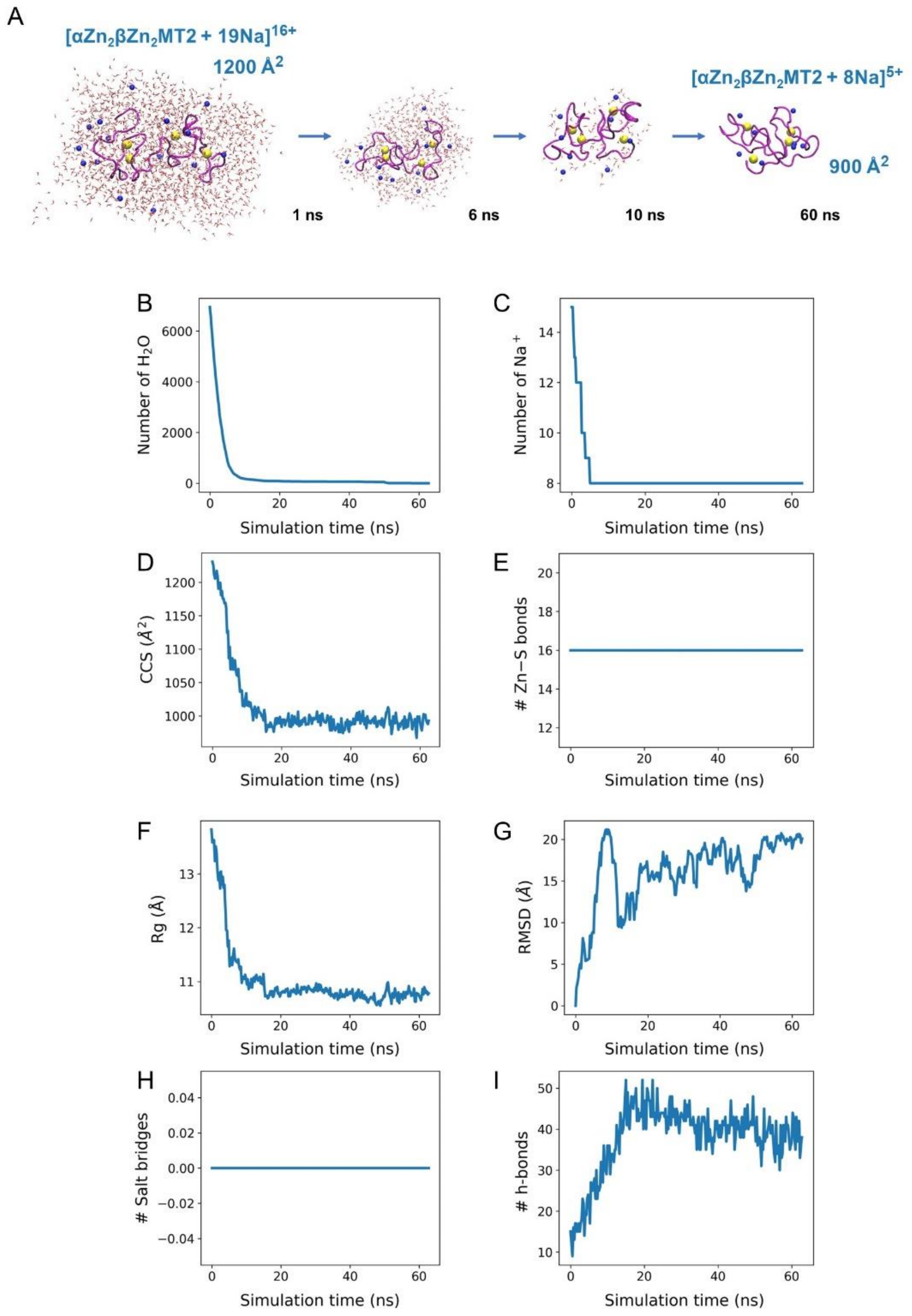

Figure S6. Gas-phase MD simulations of the electrospray ionization process of aqueous nanodroplet containing αZn$_2$βZn$_2$MT2 and Na$^+$ to achieve a 16+ overall charge. (A) Snapshots of the desolvation process at different frames. Na$^+$ is represented by a blue sphere, the protein backbone is shown in magenta, and the oxygen atoms from solvent molecules are shown in red. The number of water molecules (B), Na$^+$ ions (C), CCS values (D), number of Zn−S bonds (E), the radius of gyration (F), root-mean-square deviation (G), number of salt bridges (H) and hydrogen bonds (I) were monitored throughout the desolvation.

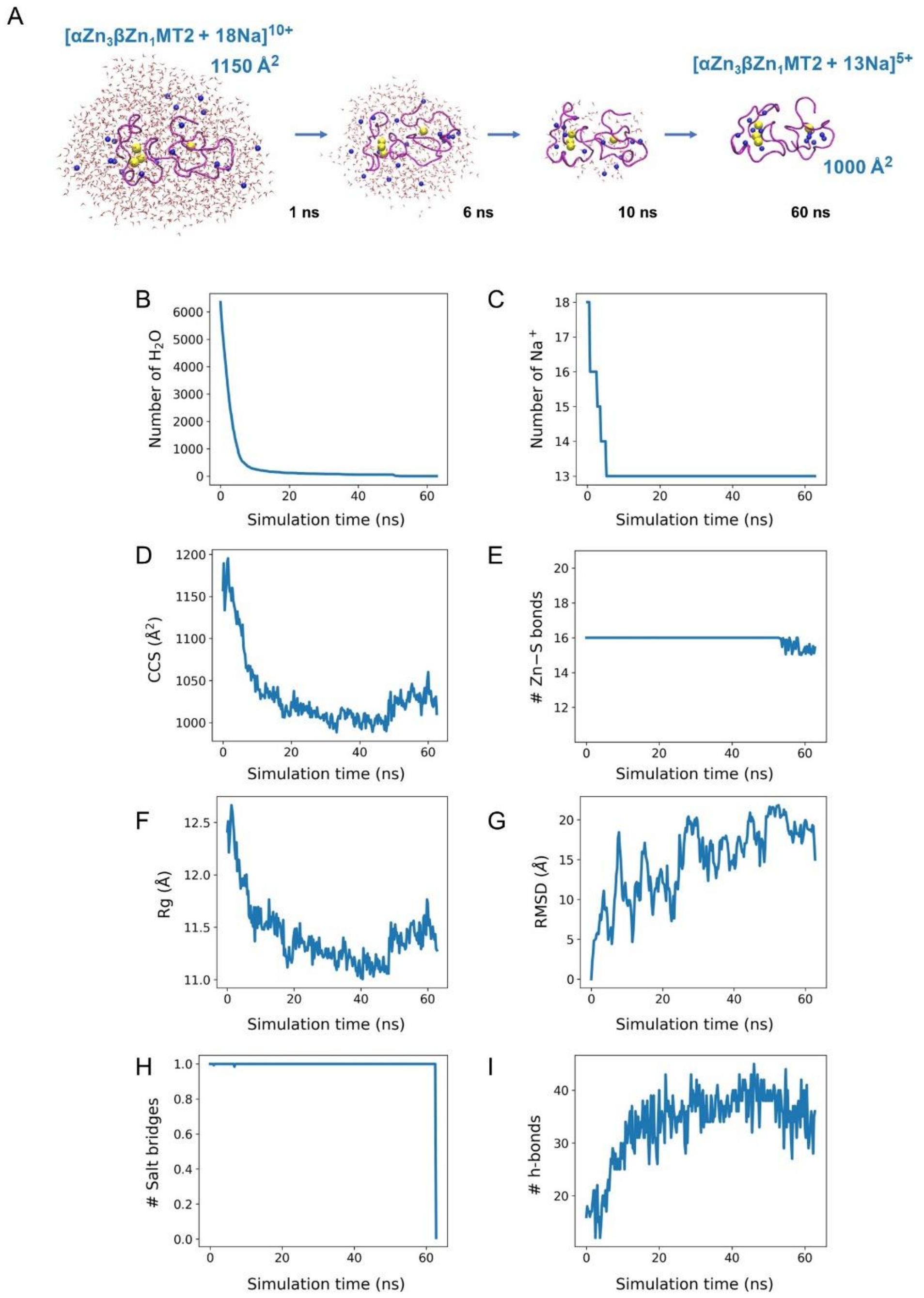

Figure S7. Gas-phase MD simulations of the electrospray ionization process of aqueous nanodroplet containing $\alpha Zn_3\beta Zn_1$MT2 and Na^+ to achieve a 16+ overall charge. (A) Snapshots of the desolvation process at different frames. Na^+ is represented by a blue sphere, the protein backbone is shown in magenta, and the oxygen atoms from solvent molecules are shown in red. The number of water molecules (B), Na^+ ions (C), CCS values (D), number of Zn−S bonds (E), the radius of gyration (F), root-mean-square deviation (G), number of salt bridges (H) and hydrogen bonds (I) were monitored throughout the desolvation.

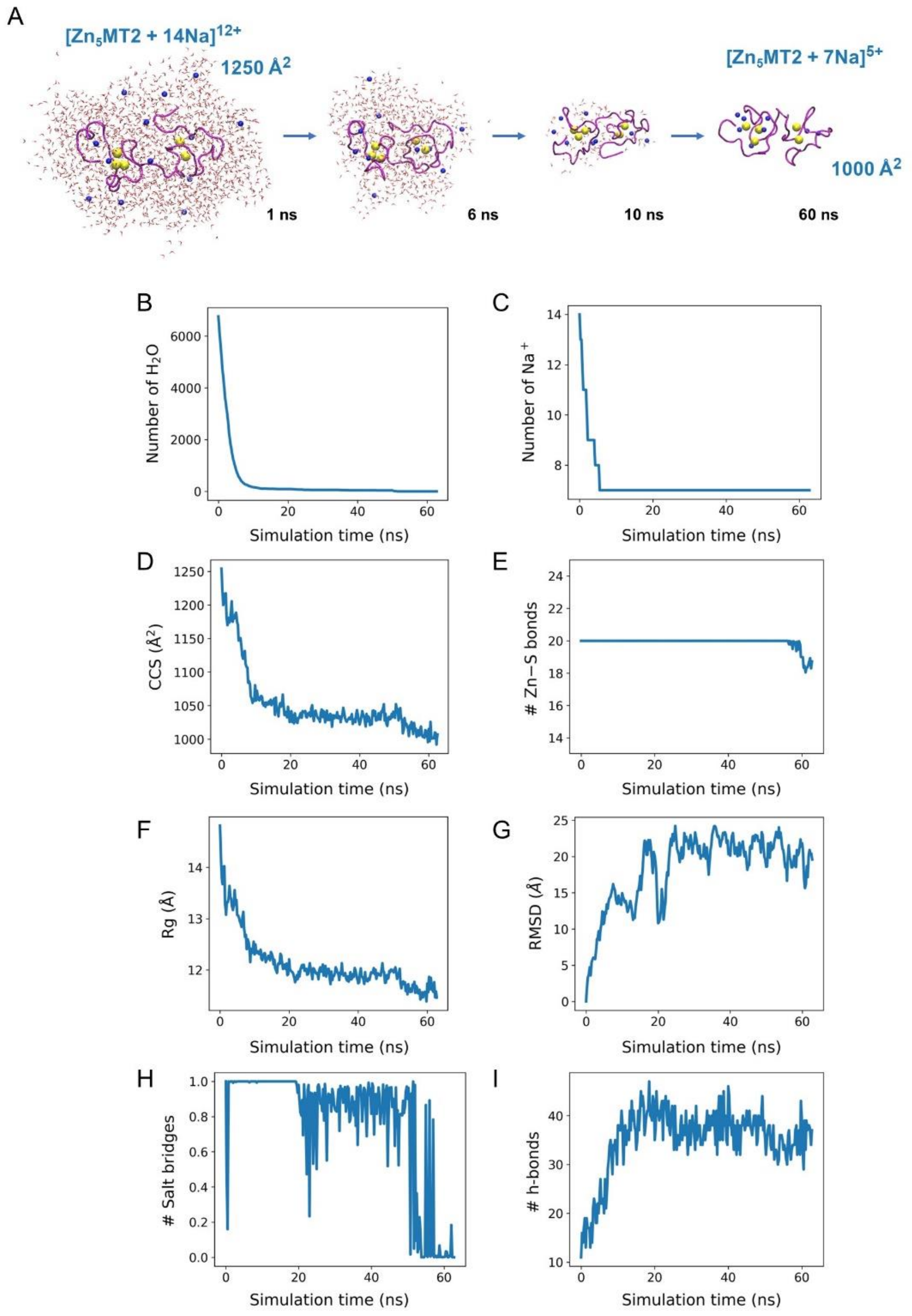

Figure S8. Gas-phase MD simulations of the electrospray ionization process of aqueous nanodroplet containing Zn_5MT2 and Na^+ to achieve a 16+ overall charge. (A) Snapshots of the desolvation process at different frames. Na^+ is represented by a blue sphere, the protein backbone is shown in magenta, and the oxygen atoms from solvent molecules are shown in red. The number of water molecules (B), Na^+ ions (C), CCS values (D), number of Zn─S bonds (E), the radius of gyration (F), root-mean-square deviation (G), number of salt bridges (H) and hydrogen bonds (I) were monitored throughout the desolvation.

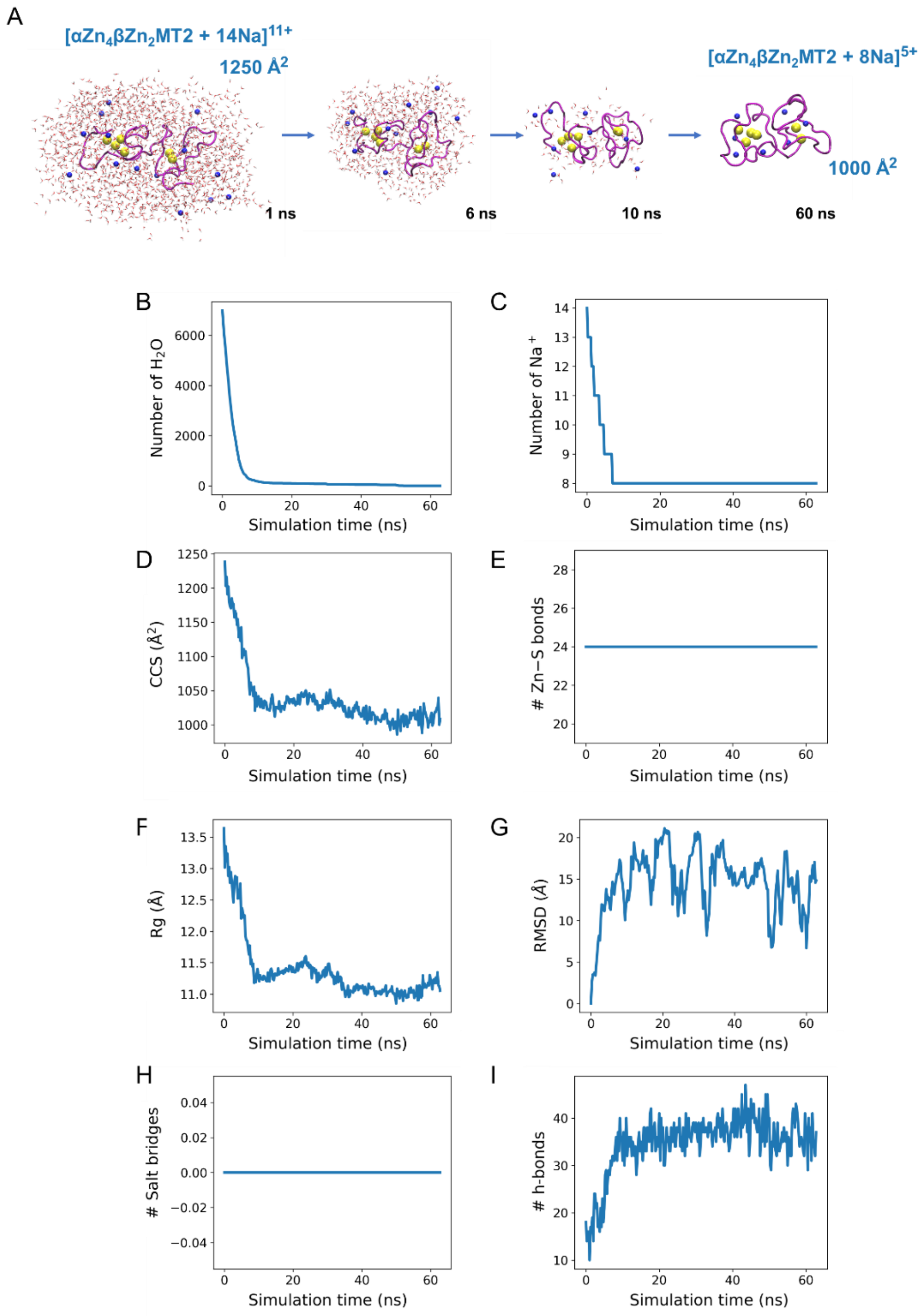

Figure S9. Gas-phase MD simulations of the electrospray ionization process of aqueous nanodroplet containing $\alpha Zn_4\beta Zn_2MT2$ and Na^+ to achieve a 16+ overall charge. (A) Snapshots of the desolvation process at different frames. Na^+ is represented by a blue sphere, the protein backbone is shown in magenta, and the oxygen atoms from solvent molecules are shown in red. The number of water molecules (B), Na^+ ions (C), CCS values (D), number of Zn−S bonds (E), the radius of gyration (F), root-mean-square deviation (G), number of salt bridges (H) and hydrogen bonds (I) were monitored throughout the desolvation.

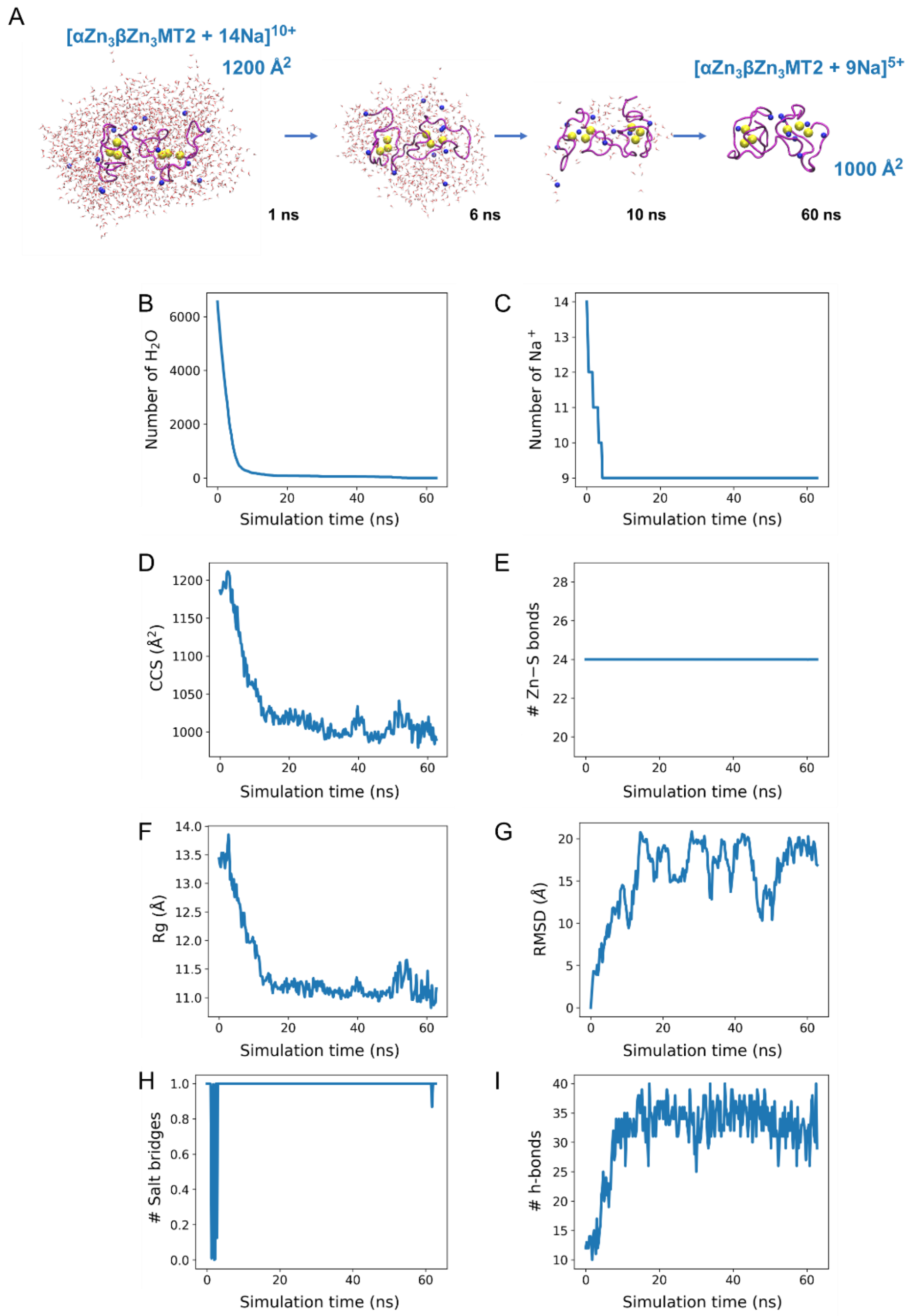

Figure S10. Gas-phase MD simulations of the electrospray ionization process of aqueous nanodroplet containing αZn3βZn3MT2 and Na^+ to achieve a 16+ overall charge. (A) Snapshots of the desolvation process at different frames. Na^+ is represented by a blue sphere, the protein backbone is shown in magenta, and the oxygen atoms from solvent molecules are shown in red. The number of water molecules (B), Na^+ ions (C), CCS values (D), number of Zn−S bonds (E), the radius of gyration (F), root-mean-square deviation (G), number of salt bridges (H) and hydrogen bonds (I) were monitored throughout the desolvation.

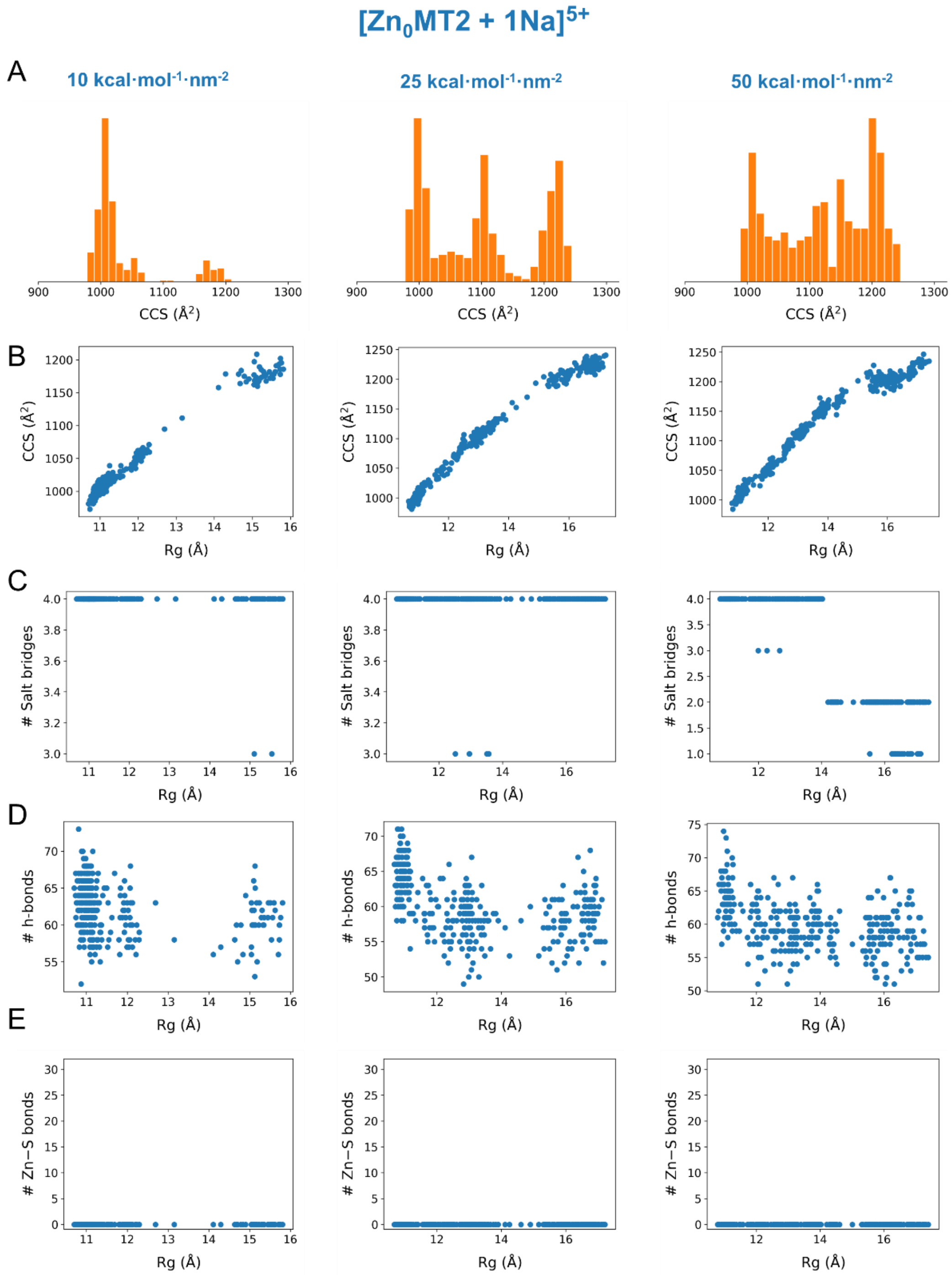

Figure S11. Optimization of the SMD simulations involving gas-phase [Zn_0MT2 +1Na]$^{5+}$ ions using the radius of gyration (R_g) as a collective variable. CCS histograms for three different force constants (10, 25, and 50 kcal·mol^{-1}·nm^{-2}) (A), CCS (B), number of salt bridges (C), number of hydrogen bonds (D) and number of Zn−S bonds (E) as a function of the R_g.

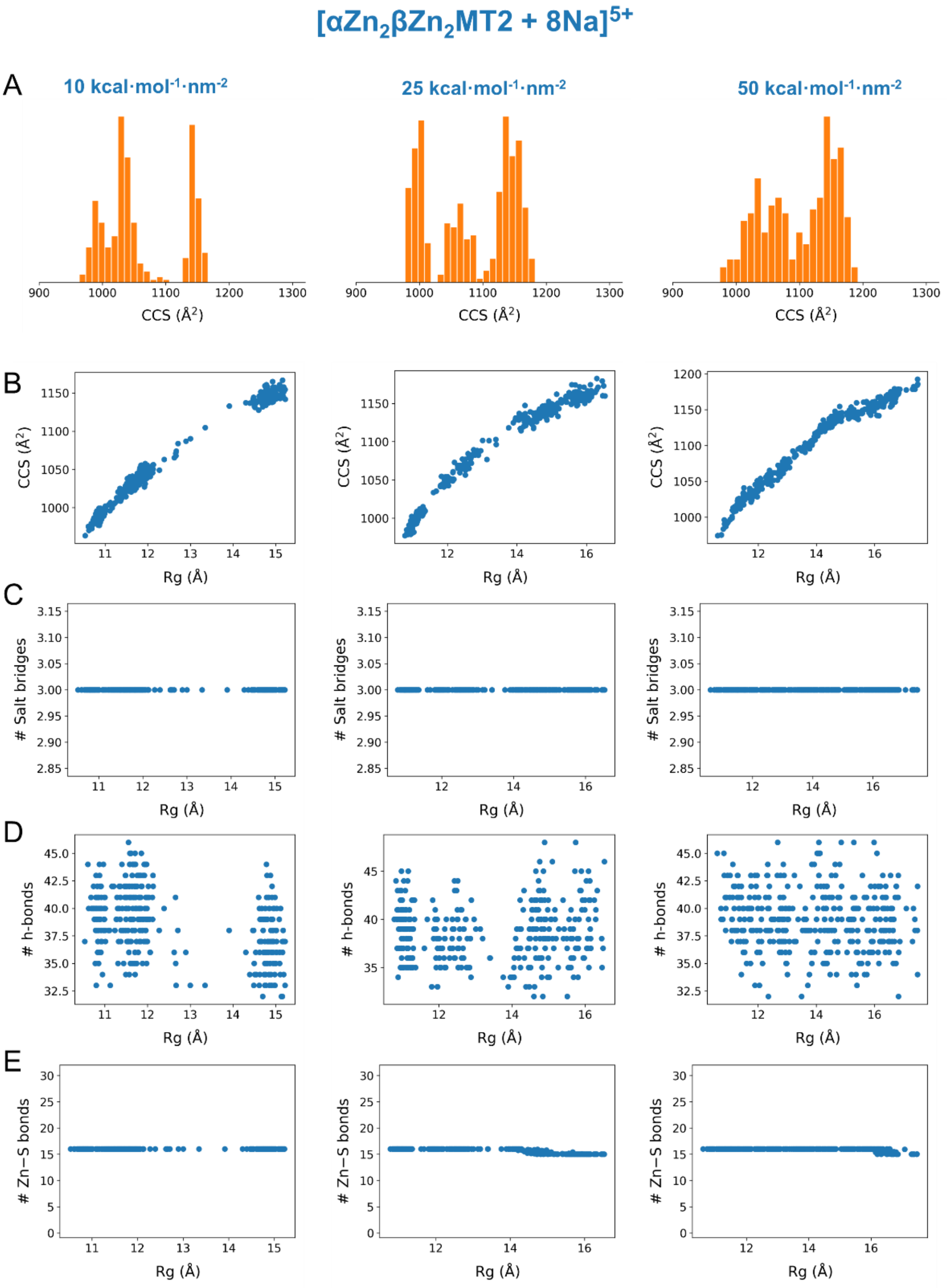

Figure S12. Optimization of the SMD simulations involving gas-phase [$\alpha Zn_2\beta Zn_2$MT2 +8Na]$^{5+}$ ions using radius of gyration (R_g) as a collective variable. CCS histograms for three different force constants (10, 25, and 50 kcal·mol^{-1}·nm^{-2}) (A), CCS (B), number of salt bridges (C), number of hydrogen bonds (D) and number of Zn−S bonds (E) as a function of the R_g.

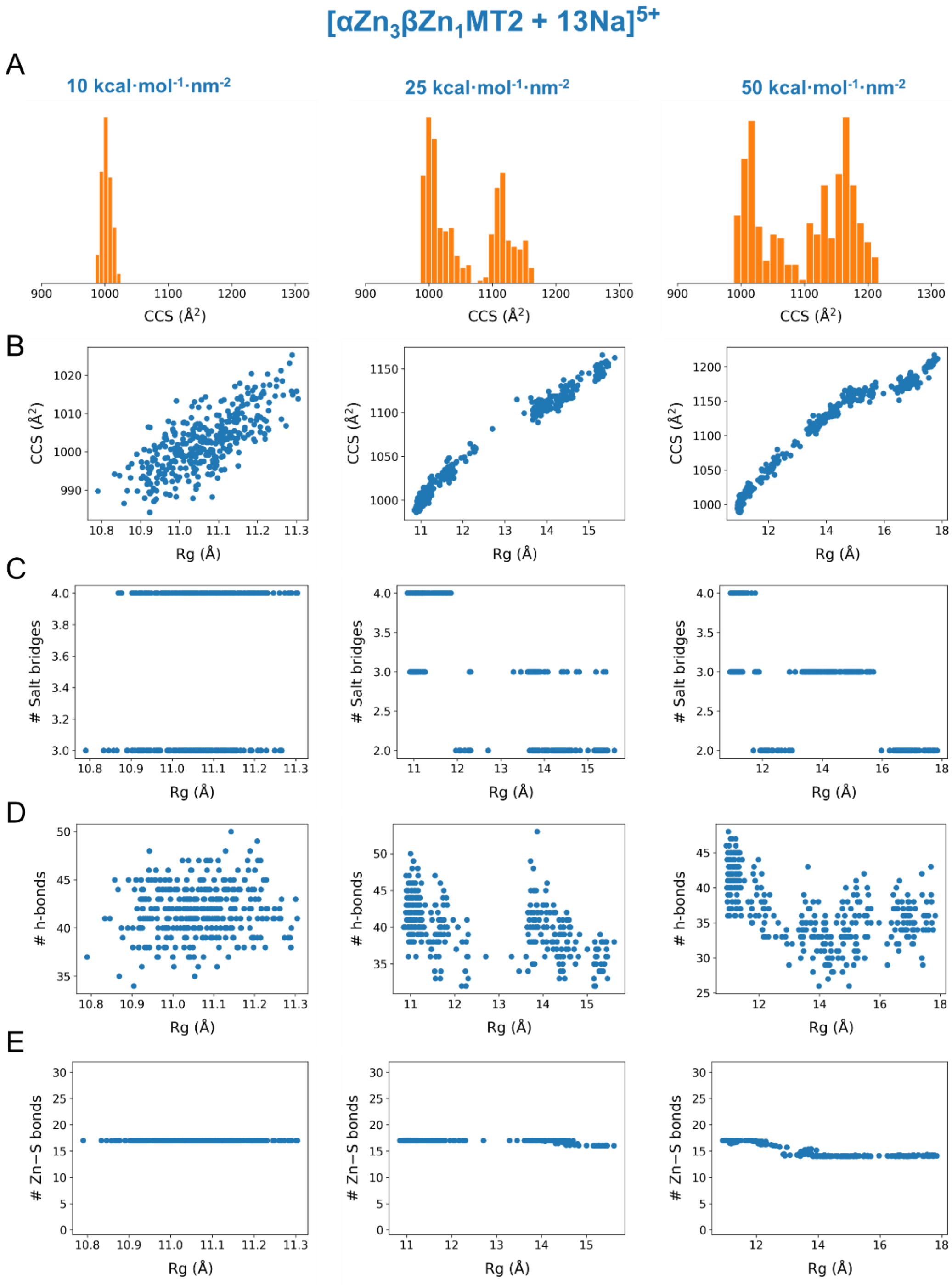

Figure S13. Optimization of the SMD simulations involving gas-phase [$\alpha Zn_3\beta Zn_1$MT2 +13Na]$^{5+}$ ions using radius of gyration (R_g) as a collective variable. CCS histograms for three different force constants (10, 25, and 50 kcal·mol^{-1}·nm^{-2}) (A), CCS (B), number of salt bridges (C), number of hydrogen bonds (D) and number of Zn−S bonds (E) as a function of the R_g.

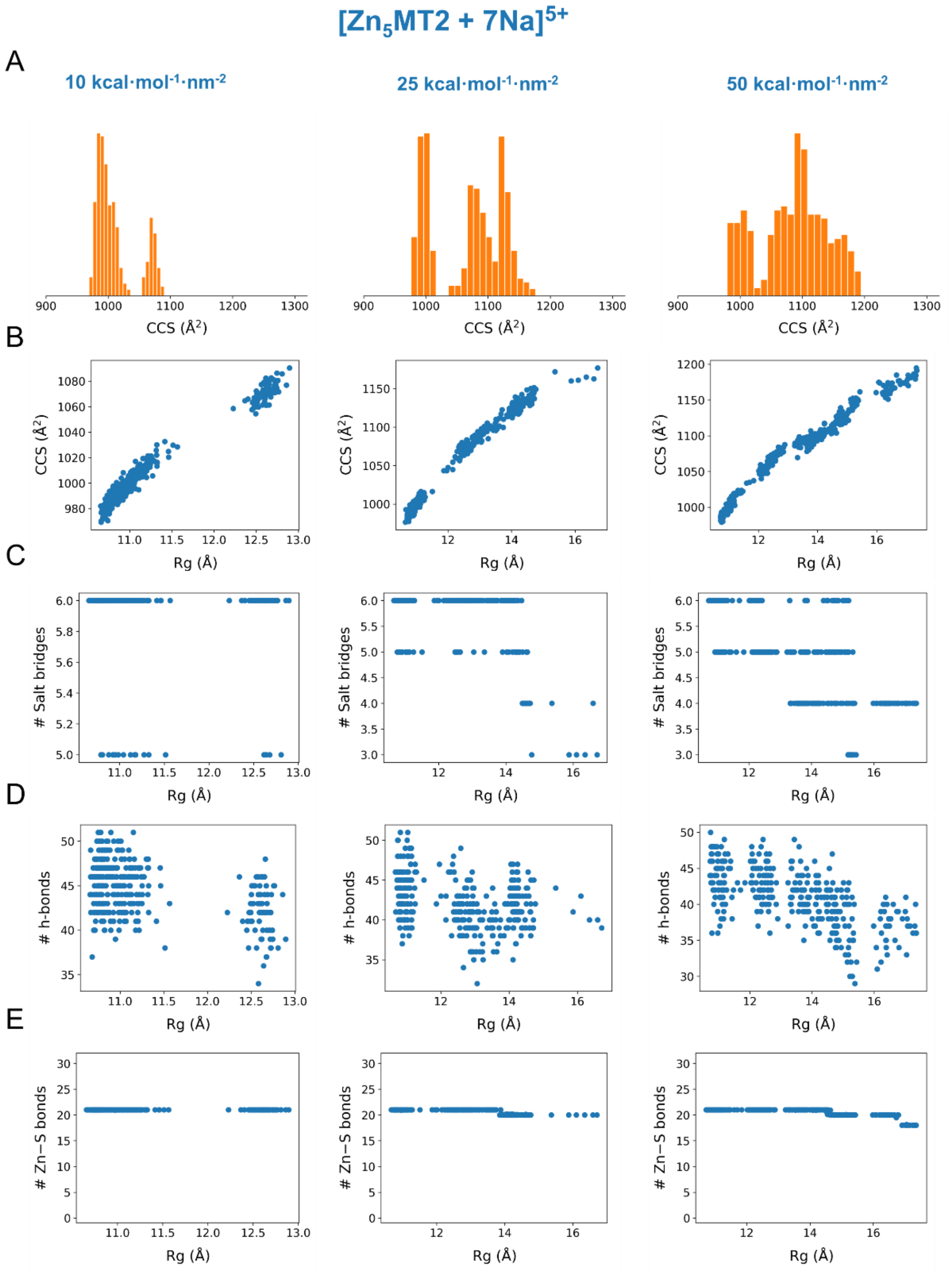

Figure S14. Optimization of the SMD simulations involving gas-phase $[Zn_5MT2 + 7Na]^{5+}$ ions using radius of gyration (R_g) as a collective variable. CCS histograms for three different force constants (10, 25, and 50 kcal·mol^{-1}·nm^{-2}) (A), CCS (B), number of salt bridges (C), number of hydrogen bonds (D) and number of Zn−S bonds (E) as a function of the R_g.

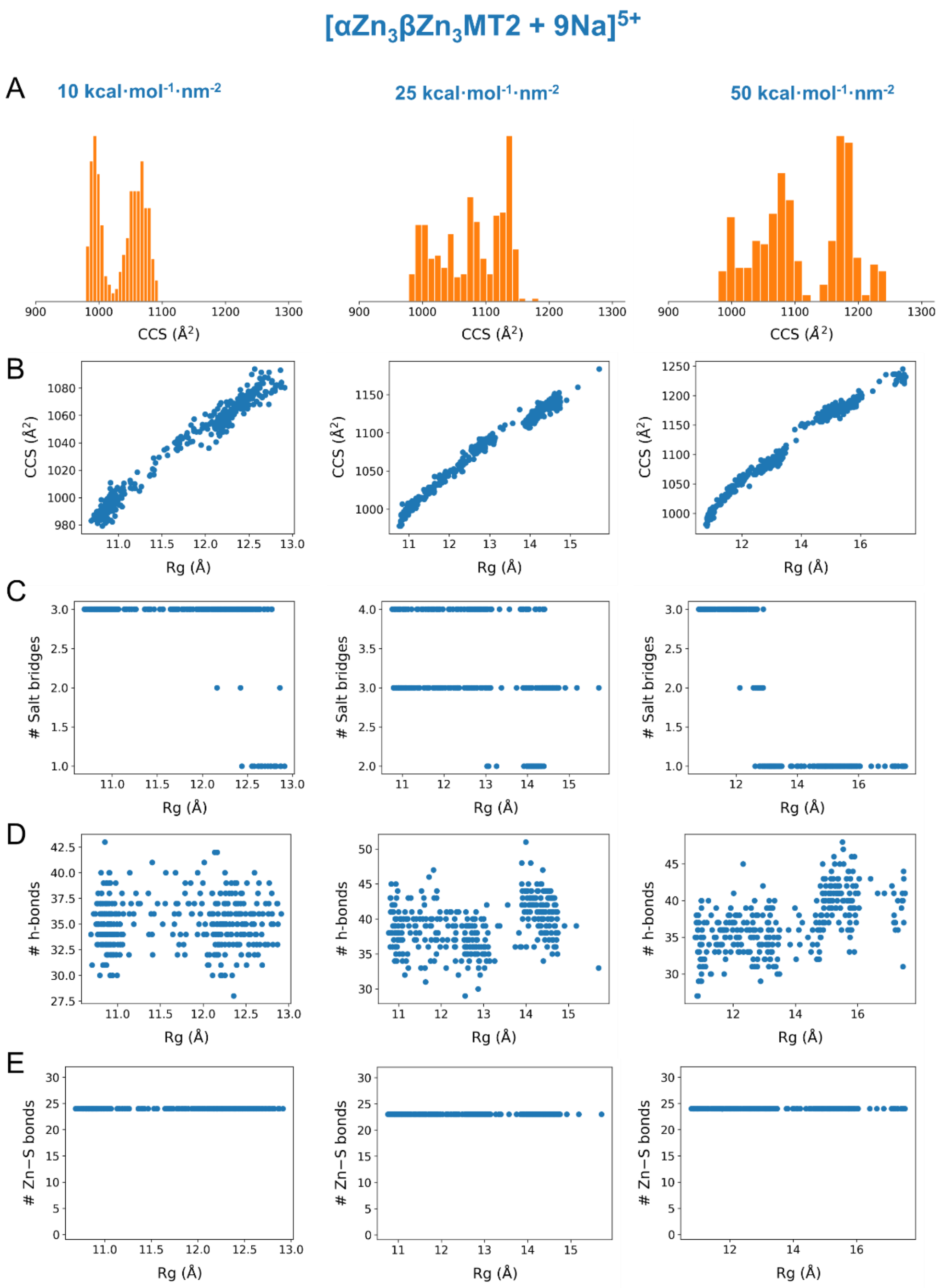

Figure S15. Optimization of the SMD simulations involving gas-phase [$\alpha Zn_3\beta Zn_3$MT2 +9Na]$^{5+}$ ions using radius of gyration (R_g) as a collective variable. CCS histograms for three different force constants (10, 25, and 50 kcal·mol^{-1}·nm^{-2}) (A), CCS (B), number of salt bridges (C), number of hydrogen bonds (D) and number of Zn−S bonds (E) as a function of the R_g.

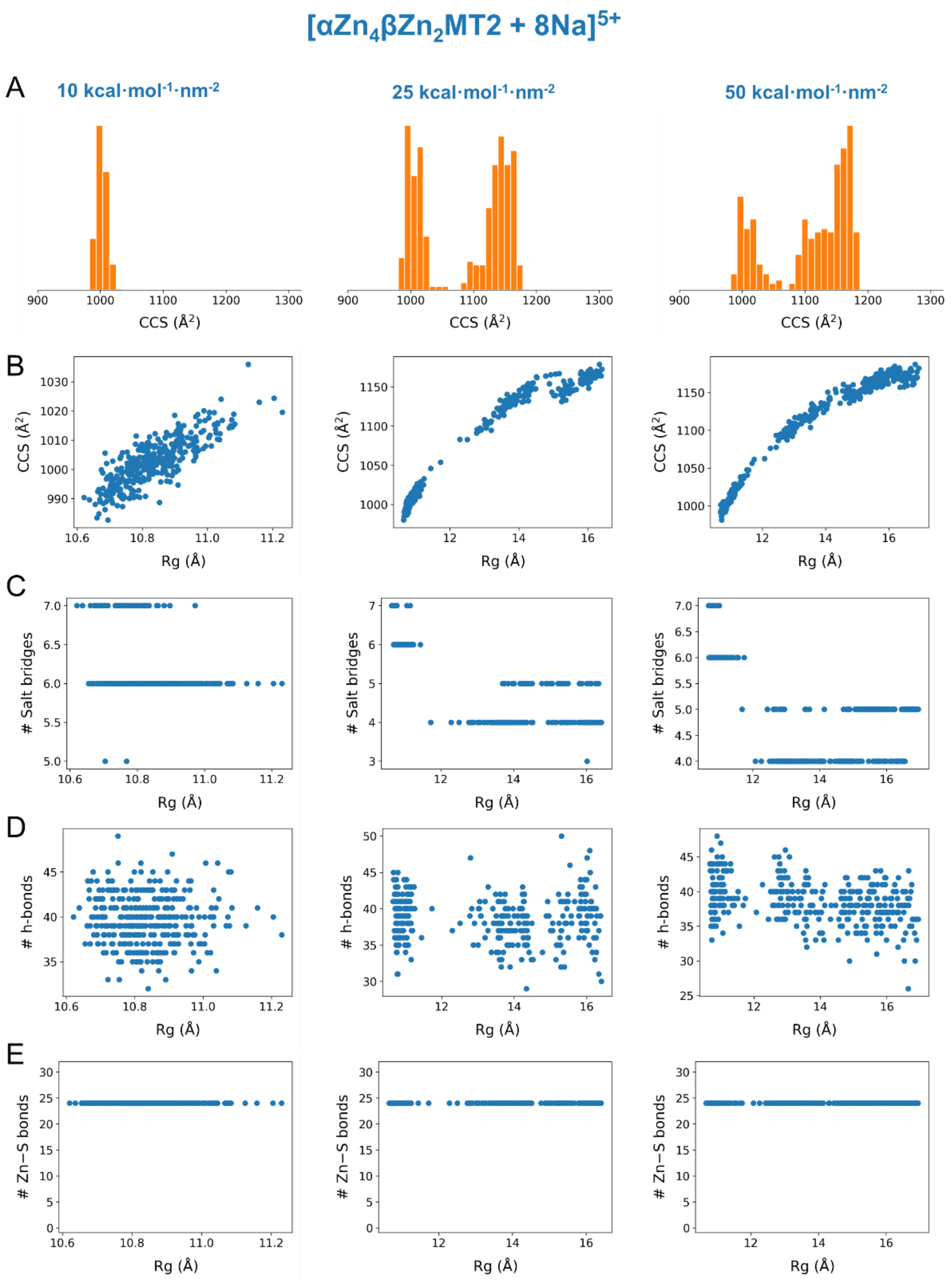

Figure S16. Optimization of the SMD simulations involving gas-phase [$\alpha Zn_4\beta Zn_2$MT2 +9Na]$^{5+}$ ions using radius of gyration (R_g) as a collective variable. CCS histograms for three different force constants (10, 25, and 50 kcal·mol^{-1}·nm^{-2}) (A), CCS (B), number of salt bridges (C), number of hydrogen bonds (D) and number of Zn−S bonds (E) as a function of the R_g.

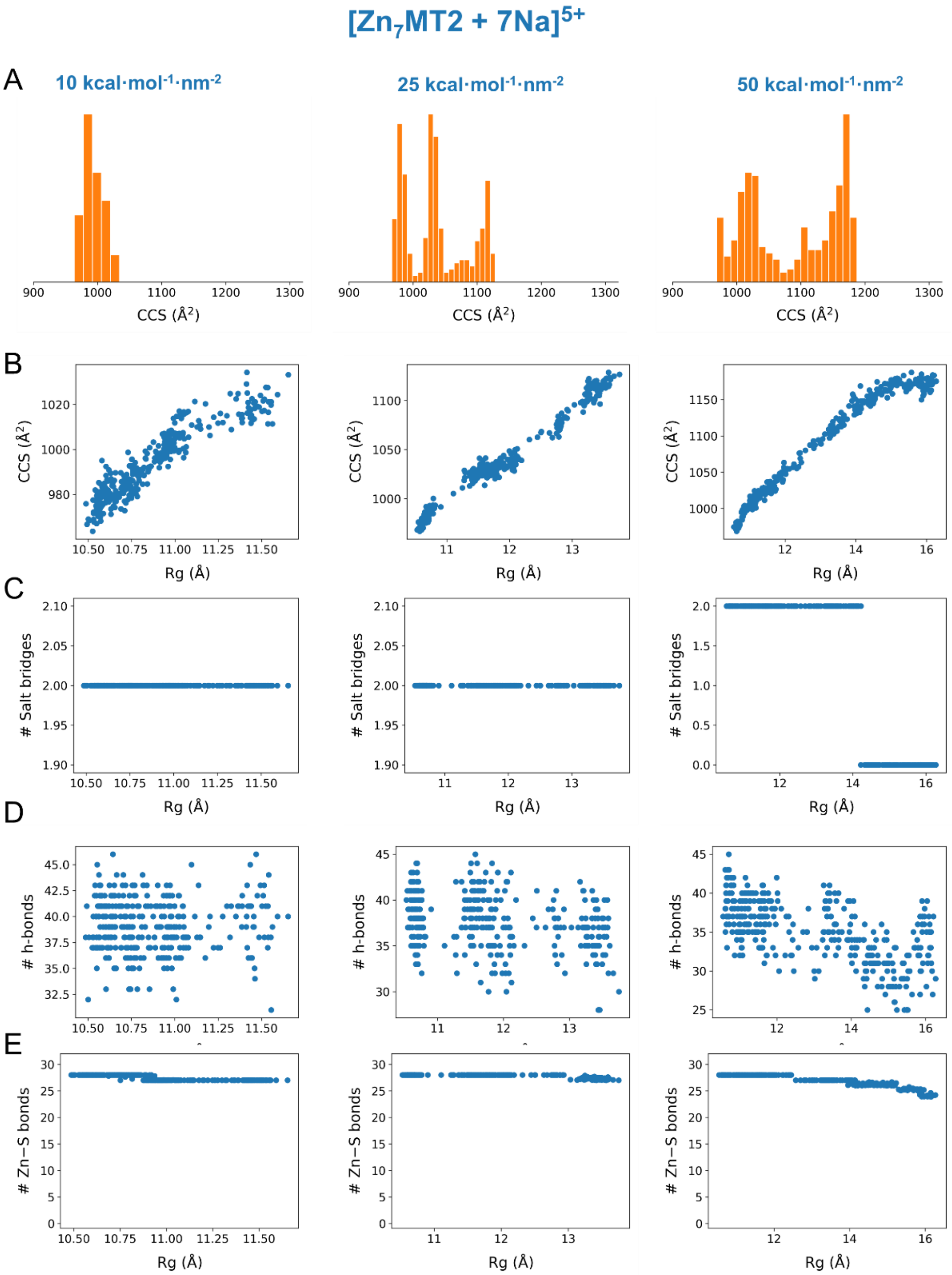

Figure S17. Optimization of the SMD simulations involving gas-phase [Zn_7MT2 +7Na]$^{5+}$ ions using radius of gyration (R_g) as a collective variable. CCS histograms for three different force constants (10, 25, and 50 kcal·mol^{-1}·nm^{-2}) (A), CCS (B), number of salt bridges (C), number of hydrogen bonds (D) and number of Zn−S bonds (E) as a function of the R_g.

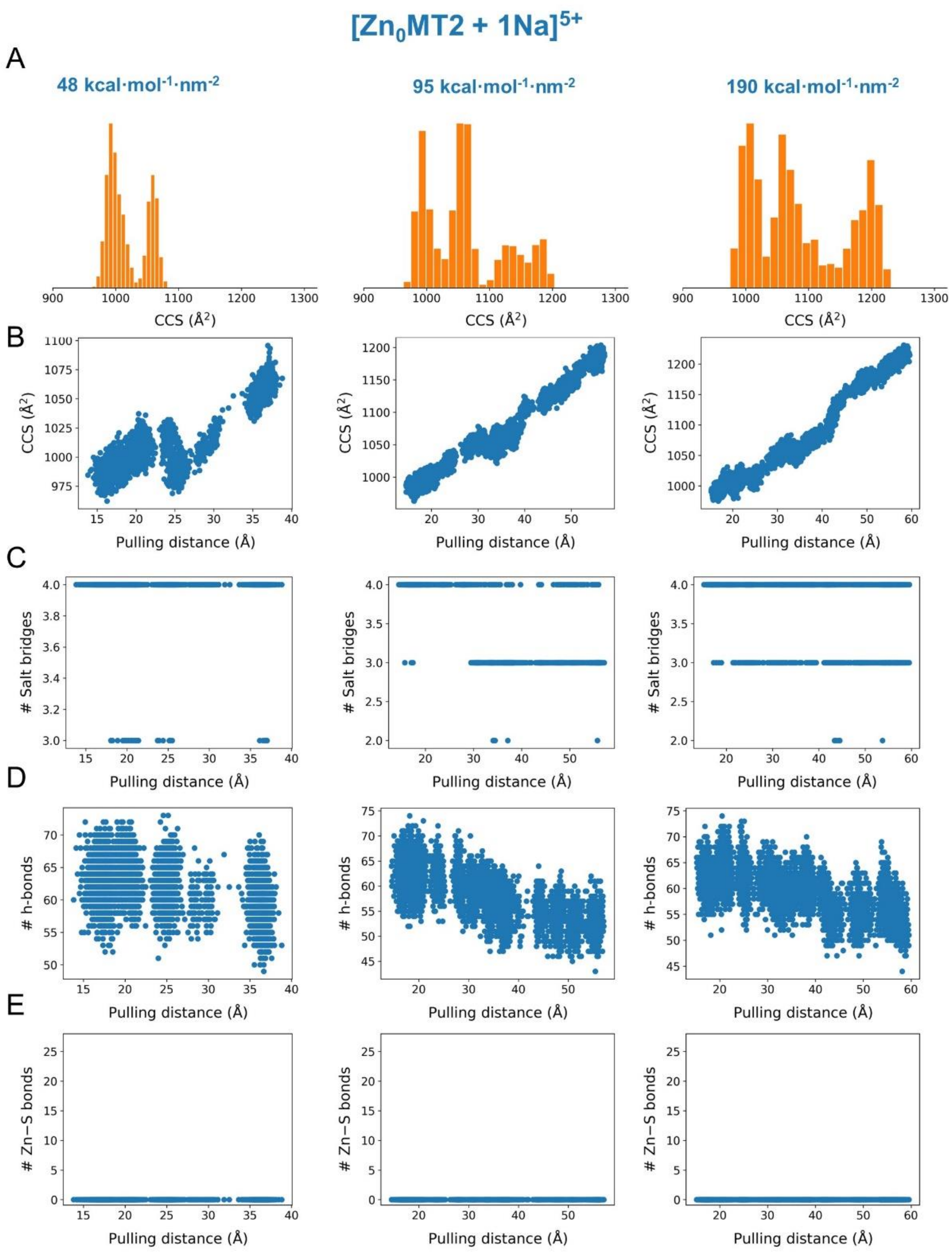

Figure S18. Optimization of the SMD simulations involving gas-phase $[Zn_0MT2 + 1Na]^{5+}$ ions using end-to-end distance (Pulling distance) as a collective variable. CCS histograms for three different force constants (48, 95, and 190 $kcal \cdot mol^{-1} \cdot nm^{-2}$) (A), CCS (B), number of salt bridges (C), number of hydrogen bonds (D) and number of Zn−S bonds (E) as a function of the Pulling distance.

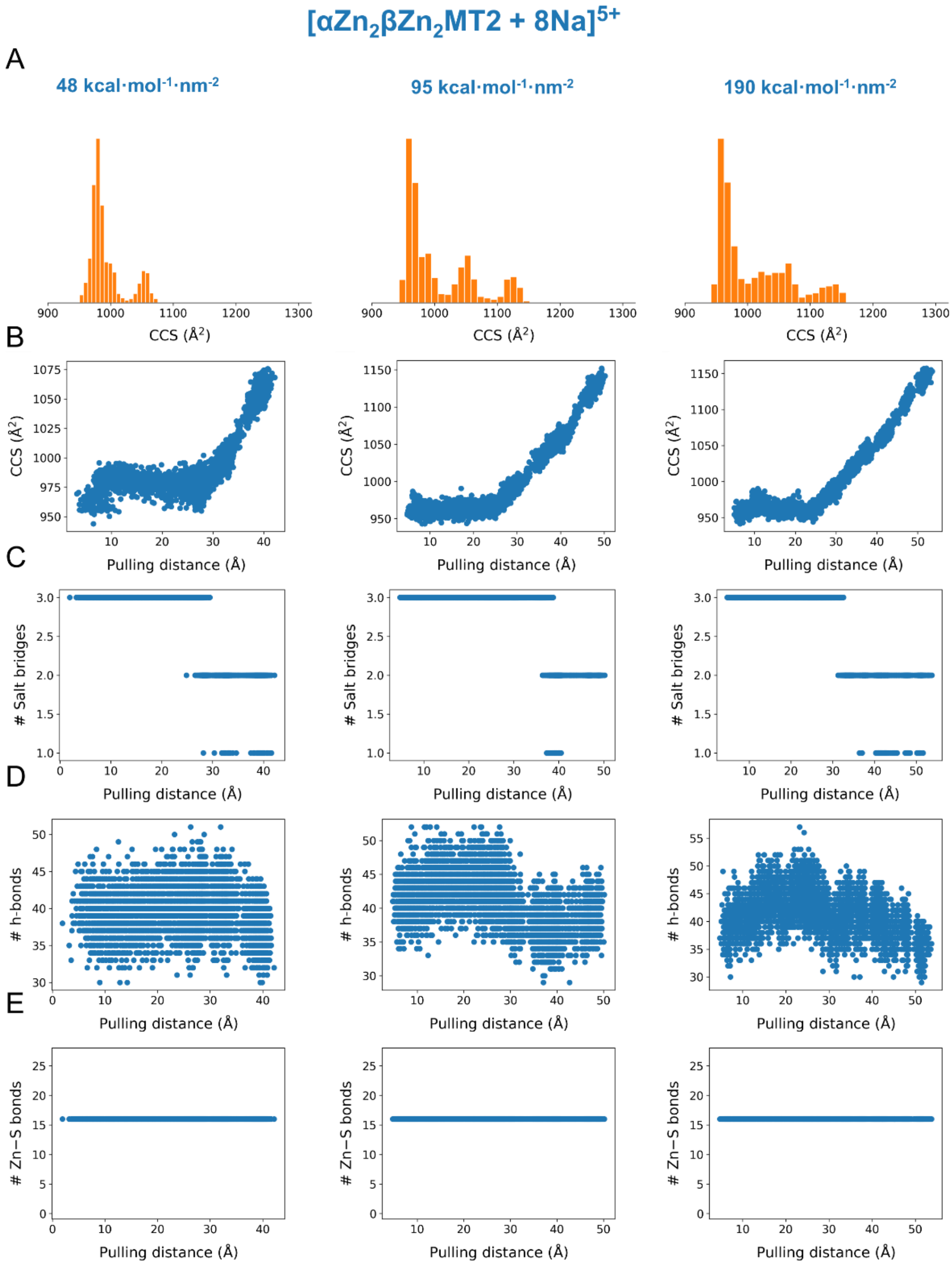

Figure S19. Optimization of the SMD simulations involving gas-phase [$\alpha Zn_2\beta Zn_2$MT2 +8Na]$^{5+}$ ions using end-to-end distance (Pulling distance) as a collective variable. CCS histograms for three different force constants (48, 95, and 190 kcal·mol^{-1}·nm^{-2}) (A), CCS (B), number of salt bridges (C), number of hydrogen bonds (D) and number of Zn−S bonds (E) as a function of the Pulling distance.

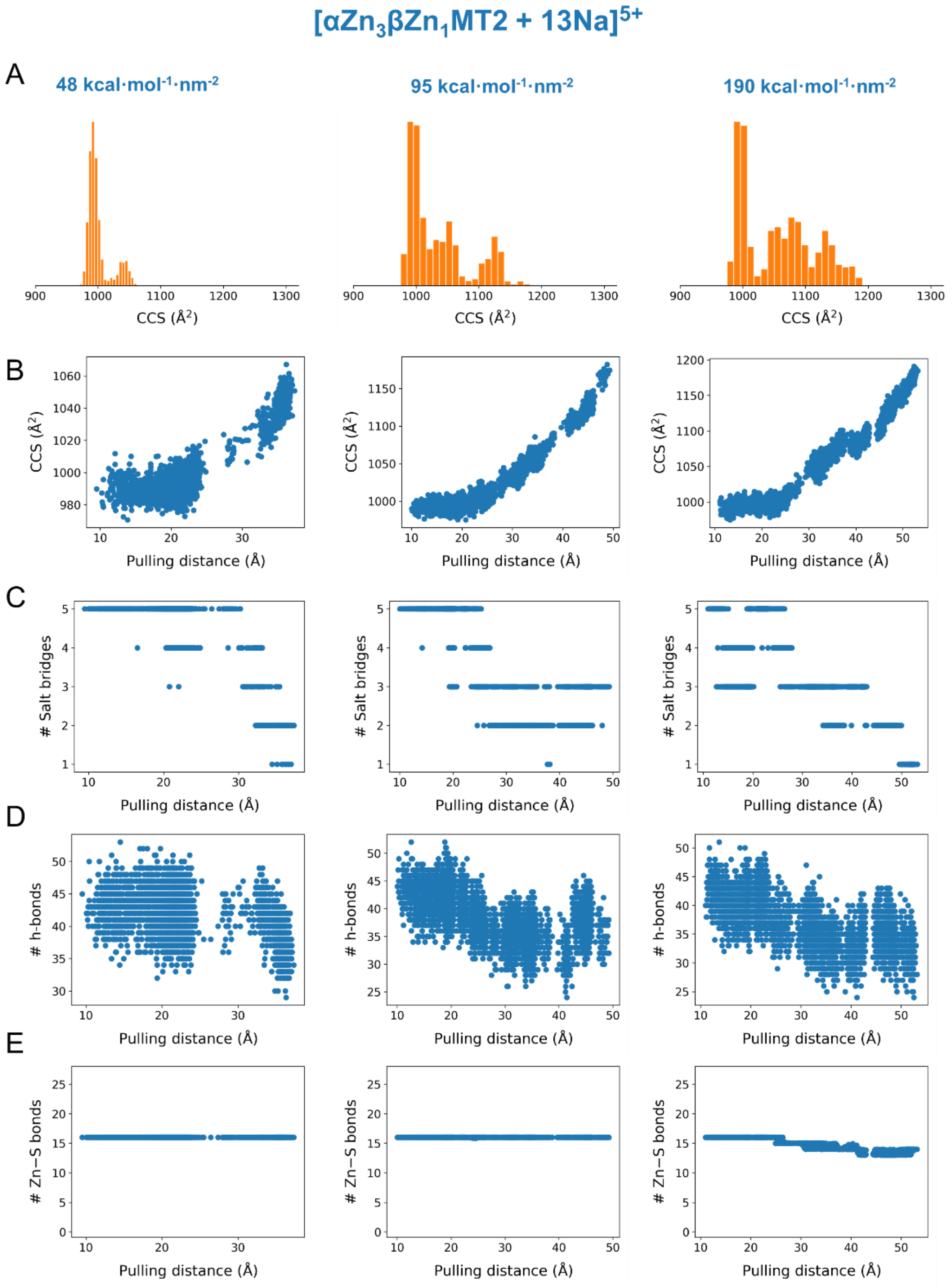

Figure S20. Optimization of the SMD simulations involving gas-phase [αZn$_3$βZn$_1$MT2 +8Na]$^{5+}$ ions using end-to-end distance (Pulling distance) as a collective variable. CCS histograms for three different force constants (48, 95, and 190 kcal·mol^{-1}·nm^{-2}) (A), CCS (B), number of salt bridges (C), number of hydrogen bonds (D) and number of Zn−S bonds (E) as a function of the Pulling distance.

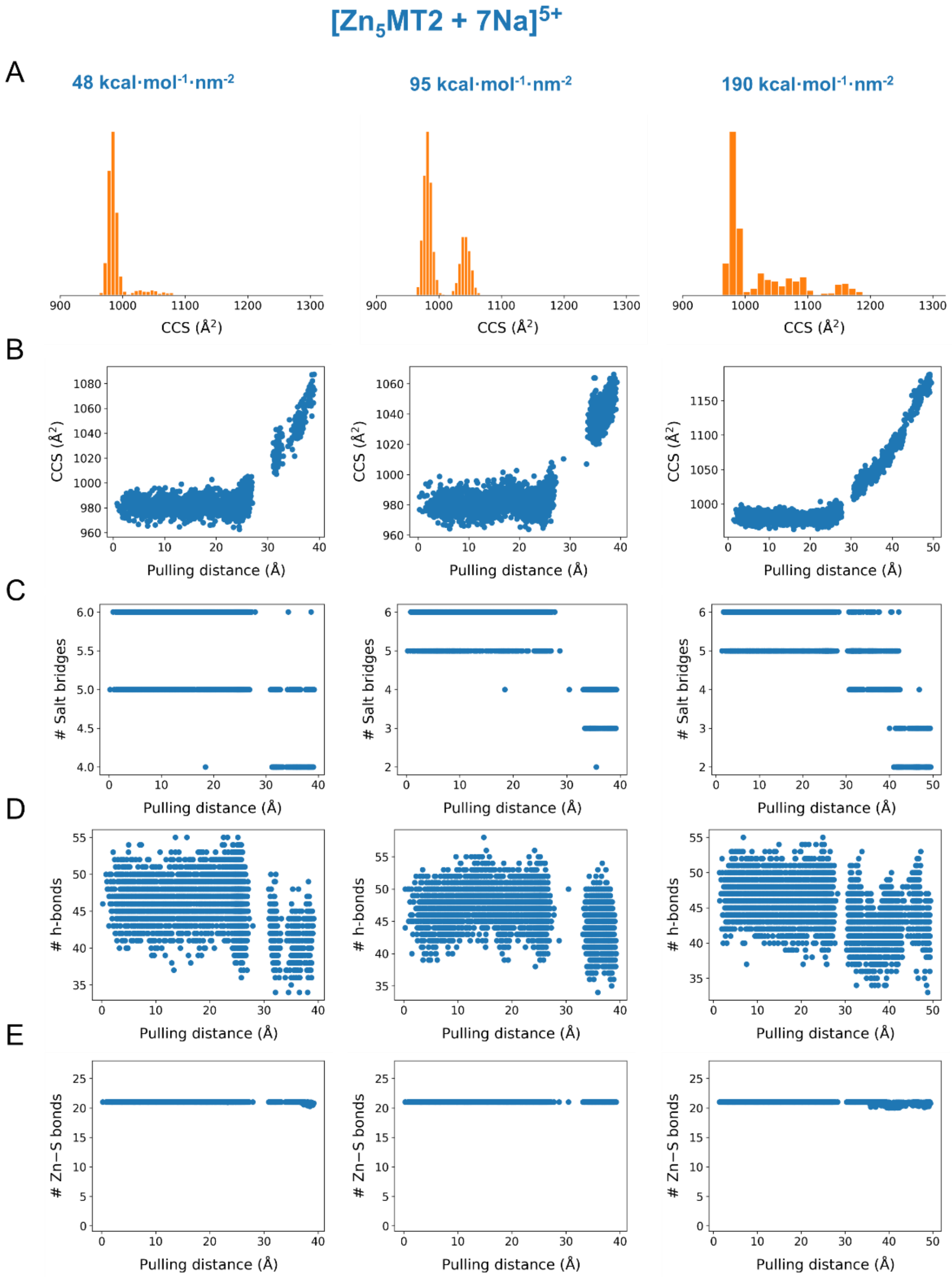

Figure S21. Optimization of the SMD simulations involving gas-phase $[Zn_5MT2 + 7Na]^{5+}$ ions using end-to-end distance (Pulling distance) as a collective variable. CCS histograms for three different force constants (48, 95, and 190 kcal·mol^{-1}·nm^{-2}) (A), CCS (B), number of salt bridges (C), number of hydrogen bonds (D) and number of Zn−S bonds (E) as a function of the Pulling distance.

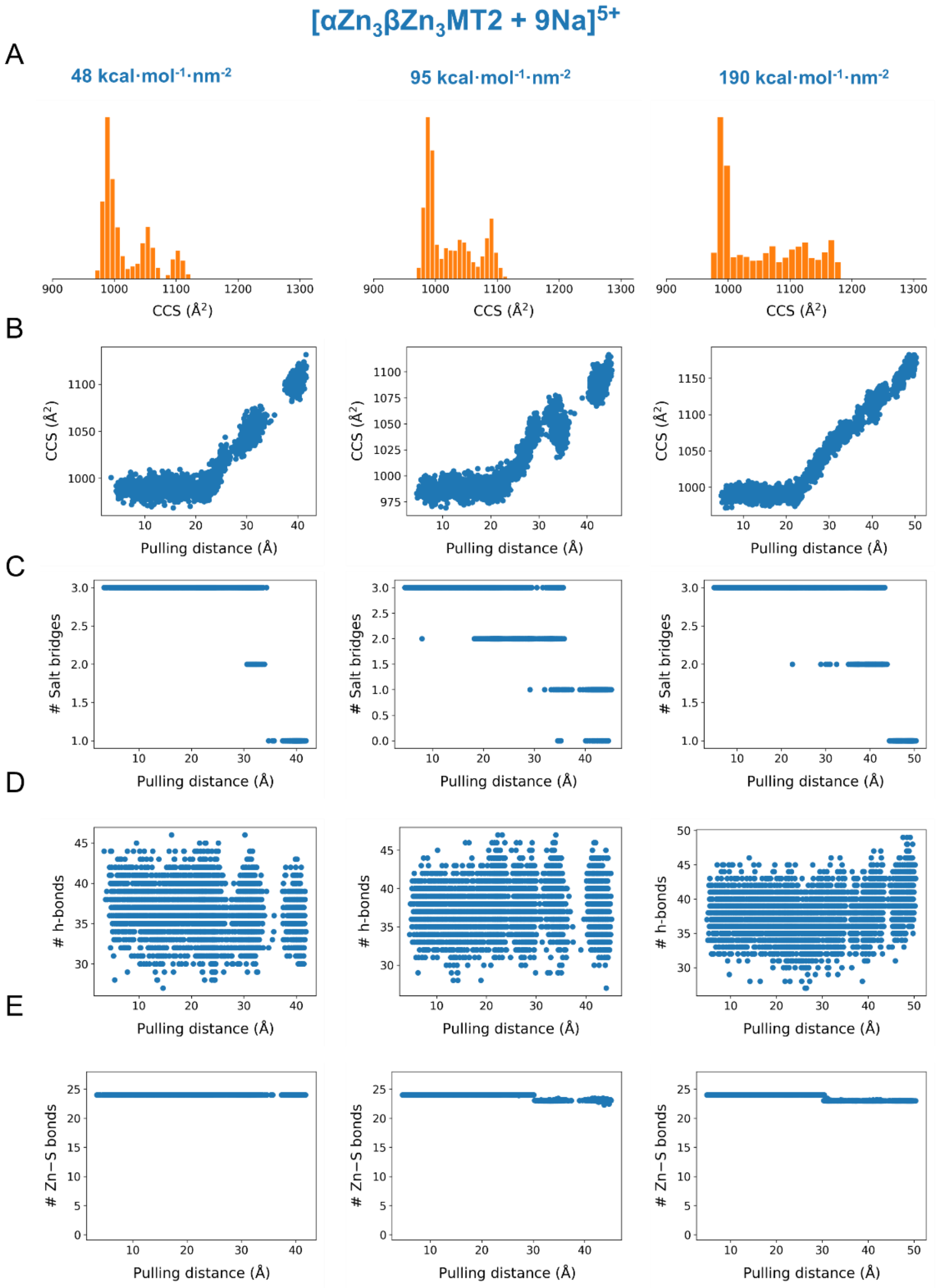

Figure S22. Optimization of the SMD simulations involving gas-phase $[\alpha Zn_3\beta Zn_3MT2 + 9Na]^{5+}$ ions using end-to-end distance (Pulling distance) as a collective variable. CCS histograms for three different force constants (48, 95, and 190 kcal·mol^{-1}·nm^{-2}) (A), CCS (B), number of salt bridges (C), number of hydrogen bonds (D) and number of Zn−S bonds (E) as a function of the Pulling distance.

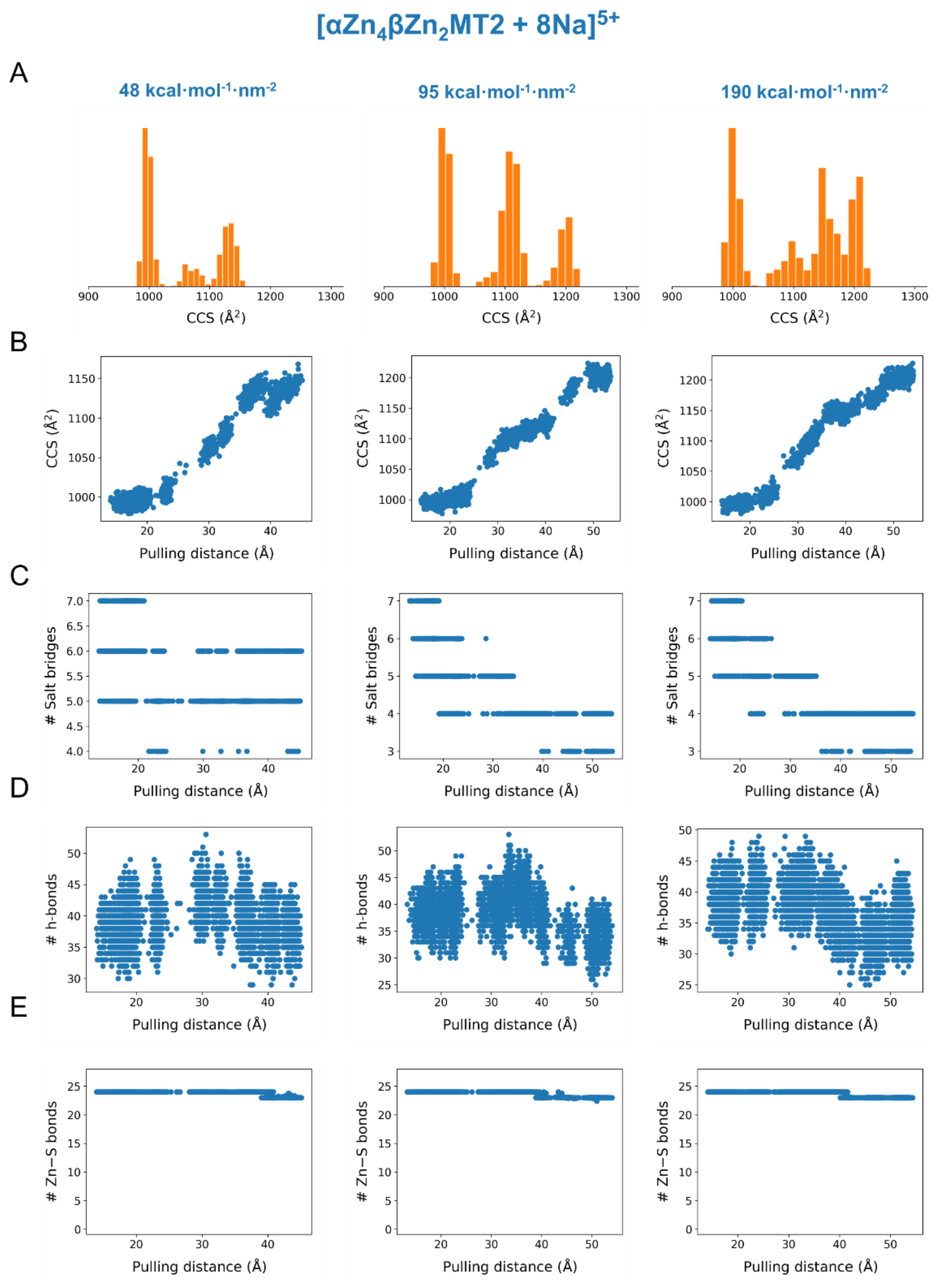

Figure S23. Optimization of the SMD simulations involving gas-phase [$\alpha Zn_4\beta Zn_2$MT2 + 8Na]$^{5+}$ ions using end-to-end distance (Pulling distance) as a collective variable. CCS histograms for three different force constants (48, 95, and 190 kcal·mol^{-1}·nm^{-2}) (A), CCS (B), number of salt bridges (C), number of hydrogen bonds (D) and number of Zn−S bonds (E) as a function of the Pulling distance.

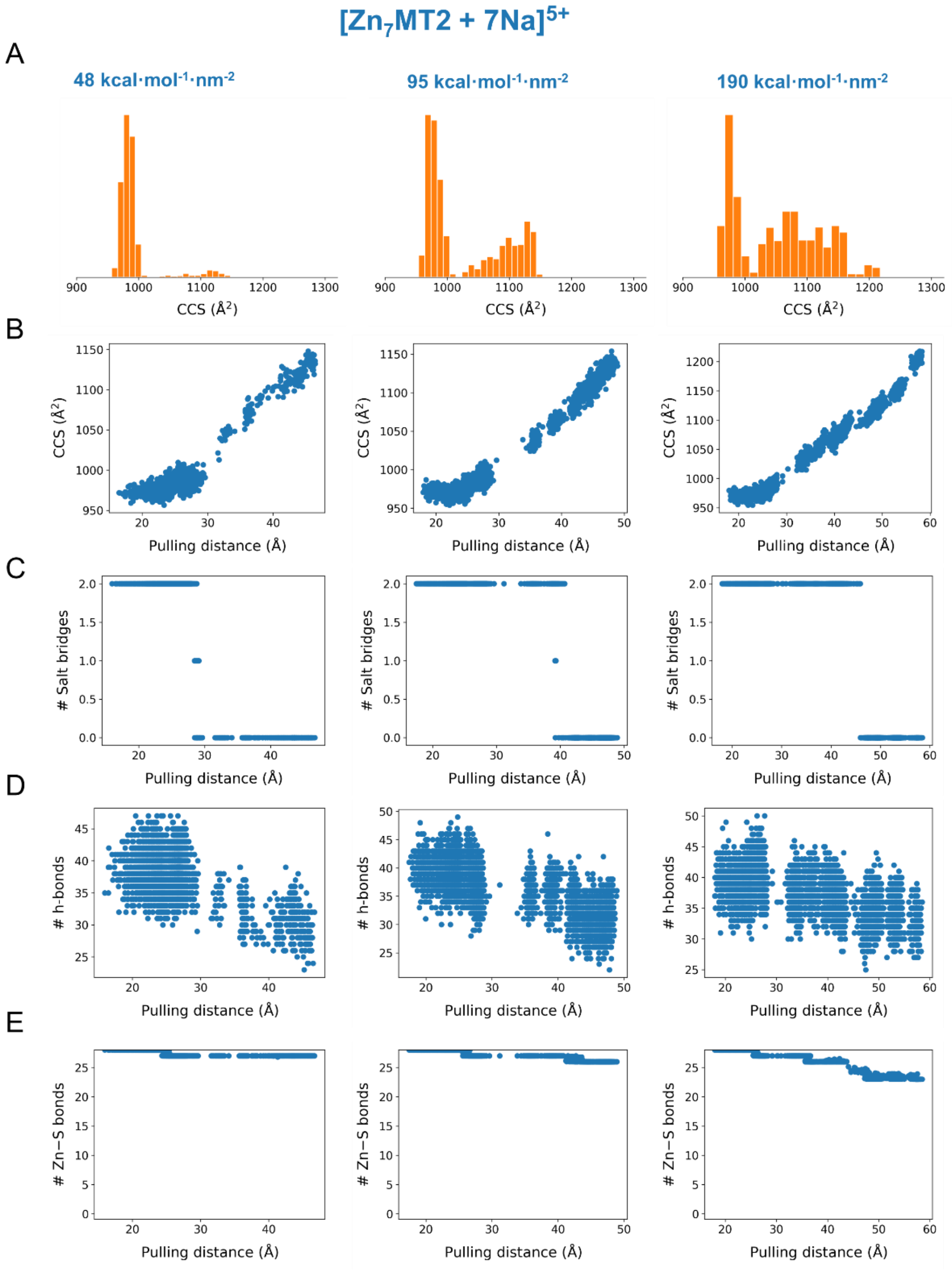

Figure S24. Optimization of the SMD simulations involving gas-phase $[Zn_7MT2 + 7Na]^{5+}$ ions using end-to-end distance (Pulling distance) as a collective variable. CCS histograms for three different force constants (48, 95, and 190 kcal·mol^{-1}·nm^{-2}) (A), CCS (B), number of salt bridges (C), number of hydrogen bonds (D) and number of Zn−S bonds (E) as a function of the Pulling distance.

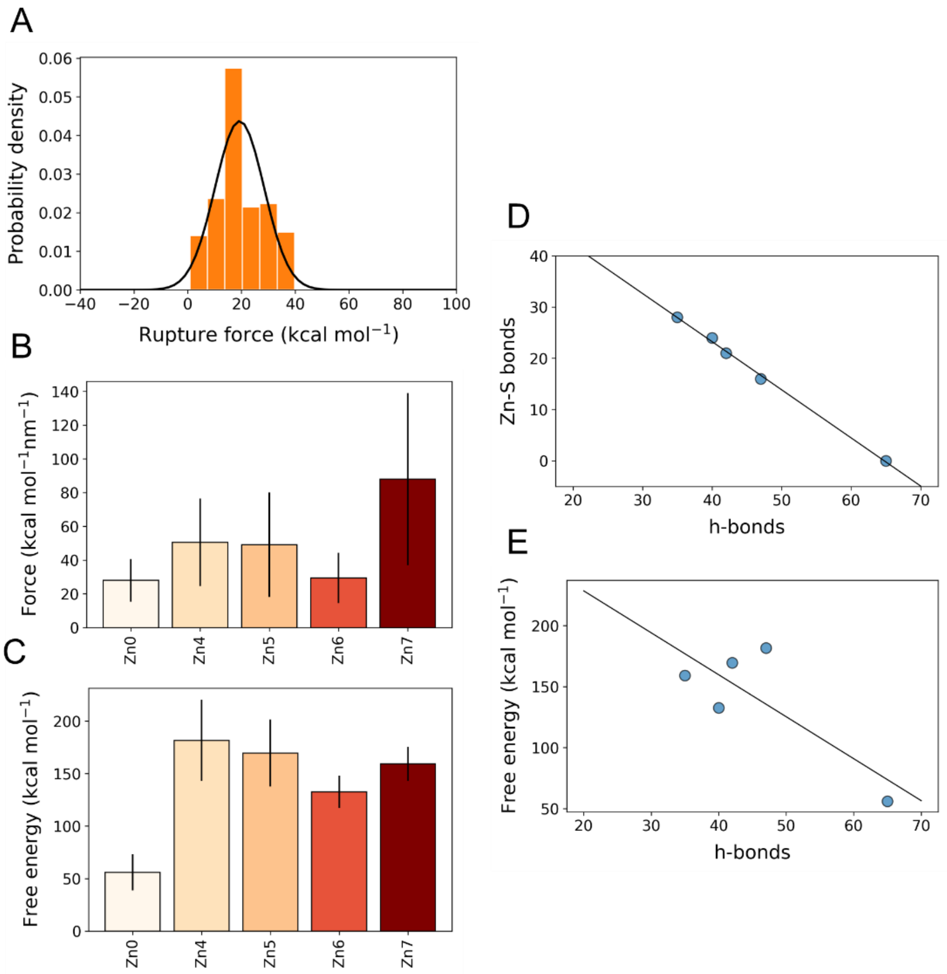

Figure S25. Analysis of the steered molecular dynamics (SMD) simulations using the radius of gyration (*R*g) as collective variable, and of the Metadynamics simulations. (A) Rupture force histogram fitted to a unimodal Gaussian distribution shown as example for apoMT2^{5+} structure. Rupture forces are the forces required to move along the collective variable R_g. (B) Bar plot representing the mean rupture or unfolding force calculated from fitting the histograms for each protein conformation studied. The black line represents the standard deviation calculated from the Gaussian distribution. (C) Bar plot representing the free energies estimated as a function of the distance between the center of masses of α- and β-domains by means well-tempered Metadynamics (WT-MetaD) simulations. The black line represents the standard deviation calculated from three independent runs. (D) Linear relationship between h-bonds and Zn–S bonds. (E) Linear relationship between h-bonds and free energy estimated by WT-MetaD.

Table S1. Accurate mass measurements of apoMT2 and Zn_{4-7}MT2 protein by native MS. The mass error corresponds to the difference between the experimental and the fitted molecular formula.

Protein	Oxidation status	formula	Mass error (Da)	$\Delta H^{+}_{(apo-ZnxMT)red}$ [a]	$\Delta H^{+}_{(apo-ZnxMT)ox}$ [b]	ΔH^{+}_{red-ox} [c]
apoMT2	reduced	$C_{223}H_{381}O_{82}N_{71}S_{21}$	0.44	0	-	-
	oxidized	$C_{223}H_{366}O_{82}N_{71}S_{21}$	0.12	-	0	15
Zn_4MT2	reduced	$C_{223}H_{371}O_{82}N_{71}S_{21}Zn_4$	0.24	10	-	-
	oxidized	$C_{223}H_{354}O_{82}N_{71}S_{21}Zn_4$	0.32	-	12	17
Zn_5MT2	reduced	$C_{223}H_{365}O_{82}N_{71}S_{21}Zn_5$	0.02	16	-	-
	oxidized	$C_{223}H_{349}O_{82}N_{71}S_{21}Zn_5$	0.25	-	17	16
Zn_6MT2	reduced	$C_{223}H_{365}O_{82}N_{71}S_{21}Zn_6$	0.22	16	-	-
	oxidized	$C_{223}H_{349}O_{82}N_{71}S_{21}Zn_6$	0.06	-	17	16
Zn_7MT2	reduced	$C_{223}H_{367}O_{82}N_{71}S_{21}Zn_7$	0.25	14	-	-
	oxidized	$C_{223}H_{359}O_{82}N_{71}S_{21}Zn_7$	0.01	-	7	8

[a]Stands for the number of protons dissociated of the reduced Zn_xMT (x = 4-7) complex with respect to reduced apoMT2. [b]Stands for the number of protons dissociated from the oxidized Zn_xMT (x = 4-7) complex with respect to oxidized apoMT2. [c]Stands for the number of protons dissociated of the reduced with respect to the oxidized Zn_xMT (x = 4-7) complex.

Table S2. Summary of the protein systems and computational methods employed in this work.

Systems	Method	Charge scheme	No. runs[a]	Simulation time (ns)
Zn_0MT2<br>$\alpha Zn_3\beta Zn_1MT2$<br>$\alpha Zn_2\beta Zn_2MT2$<br>Zn_5MT2<br>$\alpha Zn_3\beta Zn_3MT2$<br>$\alpha Zn_4\beta Zn_2MT2$<br>Zn_7MT2	cMD	mobile Na^+	3x100 ns (298K)<br>3x100 ns (798K)	4200
		mobile Na^+/static H^+	3x100 ns (298K)<br>3x100 ns (798K)	4200
Zn_7MT2		mobile H^+	2	3000
Zn_0MT2<br>$\alpha Zn_3\beta Zn_1MT2$<br>$\alpha Zn_2\beta Zn_2MT2$<br>Zn_5MT2<br>$\alpha Zn_3\beta Zn_3MT2$<br>$\alpha Zn_4\beta Zn_2MT2$<br>Zn_7MT2	desolvation	mobile Na^+	2	945
Zn_0MT2<br>$\alpha Zn_3\beta Zn_1MT2$<br>$\alpha Zn_2\beta Zn_2MT2$<br>Zn_5MT2<br>$\alpha Zn_3\beta Zn_3MT2$<br>$\alpha Zn_4\beta Zn_2MT2$<br>Zn_7MT2	SA	mobile Na^+	3	210
	SMD (CV: R_g)	mobile Na^+	25	120
	SMD (CV: N–C dis)	mobile Na^+	25	105
	WT-MetaD (CV: α-β dis)	mobile Na^+	25	420
			Total	13200

[a] Stands for the number of independent runs of each protein system. Abbreviations: cMD, classical molecular dynamics; SA, simulated annealing; SMD, steered molecular dynamics; CV, collective variable; R_g, radius of gyration; WT-MetaD, well-tempered metadynamics simulations.

REFERENCES

1 Krężel, A.; Maret, W. The bioinorganic chemistry of mammalian metallothioneins. *Chem. Rev.* **2021**, *121*, 14594–14648.

2 Eyer, P.; Worek, F.; Kiderlen, D.; Sinko, G.; Stuglin, A.; Simeon-Rudolf, V.; Reiner, E. Molar absorption coefficients for the reduced Ellman reagent: reassessment. *Anal. Biochem.* **2003**, *312*, 224–227.

3 Peris-Díaz, M. D.; Guran, R.; Domene, C.; de los Rios, V.; Zitka, O.; Adam, V.; Krężel, A. An integrated mass spectrometry and molecular dynamics simulations approach reveals the spatial organization impact of metal-binding sites on the stability of metal-depleted metallothionein-2 species. *J. Am. Chem. Soc.* **2021**, *143*, 16486–16501.

4 Peris-Díaz, M. D.; Guran, R..; Zitka, O.; Adam, V.; Krężel, A. Metal- and affinity-specific dual labeling of cysteine-rich proteins for identification of metal-binding sites. *Anal. Chem.* **2020**, *92*, 12950–12958.

5 France, A. P.; Migas, L. G.; Sinclair, E.; Bellina, B.; Barran, P. E. Using collision cross section distributions to assess the distribution of collision cross section values. *Anal. Chem.* **2020**, *92*, 4340–4348.

6 Dong, S.; Wagner, N. D.; Russell, D. H. Collision-induced unfolding of partially metalated metallothionein-2a: tracking unfolding reactions of gas-phase ions. *Anal. Chem.* **2018**, *90*, 11856–11862.

7 Konermann, L. Addressing a common misconception: ammonium acetate as neutral ph “buffer” for native electrospray mass spectrometry. *J. Am. Soc. Mass Spectrom.* **2017**, 28, 1827–1835.

8 Dudev, T.; Lim, C. Factors governing the protonation state of cysteines in proteins: An ab initio/CDM study. *J. Am. Chem. Soc.* **2002**, *124*, 6759–6766.

9 Polasky, D. A.; Dixit, S. M.; Fantin, S. M.; Ruotolo, B. T. CIUSuite 2: next-generation software for the analysis of gas-phase protein unfolding data. *Anal. Chem.* **2019**, *91*, 3147–3155.

10 Deslignière, E.; Botzanowski, T.; Diemer, H.; Cooper-Shepherd, D. A.; Wagner-Rousset, E.; Colas, O.; Béchade, G.; Giles, K.; Hernandez-Alba, O.; Beck, A.; Cianférani, S. High-resolution IMS-MS to assign additional disulfide bridge pairing in complementarity-determining regions of an igg4 monoclonal antibody. *J. Am. Soc. Mass Spectrom.* **2021**, *32*, 2505–2512.

11 Eldrid, C.; Ujma, J.; Kalfas, S.; Tomczyk, N.; Giles, K.; Morris, M.; Thalassinos, K. Gas phase stability of protein ions in a cyclic ion mobility spectrometry traveling wave device. *Anal. Chem.* **2019**, *91*, 7554–7561.

12 Eldrid, C.; Ben-Younis, A.; Ujma, J.; Britt, H.; Cragnolini, T.; Kalfas, S.; Cooper-Shepherd, D.; Tomczyk, N.; Giles, K.; Morris, M.; Akter, R.; Raleigh, D.; Thalassinos, K. Cyclic ion mobility-collision activation experiments elucidate protein behavior in the gas phase. *J. Am. Soc. Mass Spectrom.* **2021**, *32*, 1545–1552.

13 Migas, L. G.; France, A. P.; Bellina, B.; Barran, P. E. ORIGAMI: a software suite for activated ion mobility mass spectrometry (aIM-MS) applied to multimeric protein assemblies. *Int. J. Mass Spectrom.* **2018**, *427*, 20–28.

14 Humphrey, W.; Dalke, A.; Schulten, K. VMD: visual molecular dynamics. *J. Mol. Graph.* **1996**, *14*, 33–38.

15 Van Der Spoel, D.; Lindahl, E.; Hess, B.; Groenhof, G.; Mark, A. E.; Berendsen, H. J. C. GROMACS: fast, flexible, and free. journal of computational chemistry. *J. Comput. Chem.*, **2005**, *26*, 1701–1718.

16 Macchiagodena, M.; Pagliai, M.; Andreini, C.; Rosato, A.; Procacci, P. Upgrading and validation of the AMBER force field for histidine and cysteine zinc(II)-binding residues in sites with four protein ligands. *J. Chem. Inf. Model.* **2019**, *59*, 3803–3816.
17 Konermann, L.; Kim, S. Grotthuss Molecular dynamics simulations for modeling proton hopping in electrosprayed water droplets. *J. Chem. Theory Comput*. **2022**, *18*, 3781–3794.
18 McAllister, R. G.; Metwally, H.; Sun, Y.; Konermann, L. Release of native-like gaseous proteins from electrospray droplets via the charged residue mechanism: insights from molecular dynamics simulations. *J. Am. Chem. Soc.* **2015**, *137*, 12667–12676.
19 Konermann, L.; Aliyari, E.; Lee, J. H. Mobile protons limit the stability of salt bridges in the gas phase: implications for the structures of electrosprayed protein ions. *J. Phys. Chem. B* **2021**, *1*25, 3803–3814.
20 Konermann, L. Molecular dynamics simulations on gas-phase proteins with mobile protons: inclusion of all-atom charge solvation. *J. Phys. Chem. B* **2017**, *121*, 8102–8112.
21 Bellamy-Carter, J.; O'Grady, L.; Passmore, M.; Jenner, M.; Oldham, N. J. Decoding protein gas-phase stability with alanine scanning and collision-induced unfolding ion mobility mass spectrometry. *Anal. Sens*. **2021**, *1*, 63–69.
22 Konermann, L.; Ahadi, E.; Rodriguez, A. D.; Vahidi, S. Unraveling the mechanism of electrospray ionization. *Anal. Chem*. **2013**, *85*, 2–9.
23 Fernandez De La Mora, J. Electrospray ionization of large multiply charged species proceeds via dole's charged residue mechanism. *Anal. Chem*. **2000**, *406*, 93–104.
24 Beveridge, R.; Covill, S.; Pacholarz, K. J.; Kalapothakis, J. M. D.; Macphee, C. E.; Barran, P. E. A mass-spectrometry-based framework to define the extent of disorder in proteins. *Anal. Chem*. **2014**, *86*, 10979–10991.
25 Marklund, E. G.; Degiacomi, M. T.; Robinson, C. V.; Baldwin, A. J.; Benesch, J. L. P. Collision cross sections for structural proteomics. *Structure* **2015**, *23*, 791–799.
26 Gowers, R.; Linke, M.; Barnoud, J.; Reddy, T.; Melo, M.; Seyler, S.; Domański, J.; Dotson, D.; Buchoux, S.; Kenney, I.; Beckstein, O. MDAnalysis: a python package for the rapid analysis of molecular dynamics simulations. In Proceedings of the 15th Python in Science Conference; 2016.
27 Michaud-Agrawal, N.; Denning, E. J.; Woolf, T. B.; Beckstein, O. MDAnalysis: a toolkit for the analysis of molecular dynamics simulations. *J. Comput. Chem*. **2011**, *32*, 2319–2327.
28 McGibbon, R. T.; Beauchamp, K. A.; Harrigan, M. P.; Klein, C.; Swails, J. M.; Hernández, C. X.; Schwantes, C. R.; Wang, L. P.; Lane, T. J.; Pande, V. S. MDTraj: a modern open library for the analysis of molecular dynamics trajectories. *Biophys. J*. **2015**, *109*, 1528–1532.
29 Roe, D. R.; Cheatham, T. E. PTRAJ and CPPTRAJ: software for processing and analysis of molecular dynamics trajectory data. *J. Chem. Theory Comput.* **2013**, *9*, 3084–3095.
30 The PLUMED consortium. Promoting transparency and reproducibility in enhanced molecular simulations. *Nature Methods*. **2019**, *16*, 670–673.
31 Barducci, A.; Bussi, G.; Parinello, M. Well-tempered metadynamics: a smoothly converging and tunable free-energy method. *Phys. Rev. Lett*. **2008**, *100*, 020603.
32 Invernizzi, M.; Parinello, M. Exploration vs convergence speed in adaptive-bias enhanced sampling. *J. Chem. Theory. Comput*. **2022**, *18*, 3988–3996.

33 Evenseth, L. S. M.; Ocello, R.; Gabrielsen M.; Masetti, M.; Recanatini, M.; Sylte, I.; Cavalli, A. Exploring conformational dynamics of the extracellular venus flytrap domain of the $GABA_B$ receptor: a path-metadynamics study. *J. Chem. Inf. Model.* **2020**, *60*, 2294–2303.
